# Genome-wide investigation of transcription factor footprints and dynamics using cFOOT-seq

**DOI:** 10.1093/procel/pwaf071

**Published:** 2025-08-04

**Authors:** Heng Wang, Ang Wu, Meng-Chen Yang, Di Zhou, Xiyang Chen, Zhifei Shi, Yiqun Zhang, Yu-Xin Liu, Kai Chen, Xiaosong Wang, Xiao-Fang Cheng, Baodan He, Yutao Fu, Lan Kang, Yujun Hou, Kun Chen, Shan Bian, Juan Tang, Jianhuang Xue, Chenfei Wang, Xiaoyu Liu, Jiejun Shi, Shaorong Gao, Jia-Min Zhang

**Affiliations:** Key Laboratory of Spine and Spinal Cord Injury Repair and Regeneration of the Ministry of Education, Department of Orthopedics, Tongji Hospital, School of Life Sciences and Technology, Tongji University, Shanghai 200065, China; Shanghai Key Laboratory of Maternal Fetal Medicine, Shanghai Institute of Maternal-Fetal Medicine and Gynecologic Oncology, Clinical and Translation Research Center, Shanghai First Maternity and Infant Hospital, School of Life Sciences and Technology, Tongji University, Shanghai 200092, China; Key Laboratory of Spine and Spinal Cord Injury Repair and Regeneration of the Ministry of Education, Department of Orthopedics, Tongji Hospital, School of Life Sciences and Technology, Tongji University, Shanghai 200065, China; Key Laboratory of Spine and Spinal Cord Injury Repair and Regeneration of the Ministry of Education, Department of Orthopedics, Tongji Hospital, School of Life Sciences and Technology, Tongji University, Shanghai 200065, China; Key Laboratory of Spine and Spinal Cord Injury Repair and Regeneration of the Ministry of Education, Department of Orthopedics, Tongji Hospital, School of Life Sciences and Technology, Tongji University, Shanghai 200065, China; Shanghai Key Laboratory of Maternal Fetal Medicine, Shanghai Institute of Maternal-Fetal Medicine and Gynecologic Oncology, Clinical and Translation Research Center, Shanghai First Maternity and Infant Hospital, School of Life Sciences and Technology, Tongji University, Shanghai 200092, China; Shanghai Key Laboratory of Maternal Fetal Medicine, Shanghai Institute of Maternal-Fetal Medicine and Gynecologic Oncology, Clinical and Translation Research Center, Shanghai First Maternity and Infant Hospital, School of Life Sciences and Technology, Tongji University, Shanghai 200092, China; Key Laboratory of Spine and Spinal Cord Injury Repair and Regeneration of the Ministry of Education, Department of Orthopedics, Tongji Hospital, School of Life Sciences and Technology, Tongji University, Shanghai 200065, China; Key Laboratory of Spine and Spinal Cord Injury Repair and Regeneration of the Ministry of Education, Department of Orthopedics, Tongji Hospital, School of Life Sciences and Technology, Tongji University, Shanghai 200065, China; Shanghai Key Laboratory of Maternal Fetal Medicine, Shanghai Institute of Maternal-Fetal Medicine and Gynecologic Oncology, Clinical and Translation Research Center, Shanghai First Maternity and Infant Hospital, School of Life Sciences and Technology, Tongji University, Shanghai 200092, China; Key Laboratory of Spine and Spinal Cord Injury Repair and Regeneration of the Ministry of Education, Department of Orthopedics, Tongji Hospital, School of Life Sciences and Technology, Tongji University, Shanghai 200065, China; Key Laboratory of Spine and Spinal Cord Injury Repair and Regeneration of the Ministry of Education, Department of Orthopedics, Tongji Hospital, School of Life Sciences and Technology, Tongji University, Shanghai 200065, China; Key Laboratory of Spine and Spinal Cord Injury Repair and Regeneration of the Ministry of Education, Department of Orthopedics, Tongji Hospital, School of Life Sciences and Technology, Tongji University, Shanghai 200065, China; Key Laboratory of Spine and Spinal Cord Injury Repair and Regeneration of the Ministry of Education, Department of Orthopedics, Tongji Hospital, School of Life Sciences and Technology, Tongji University, Shanghai 200065, China; Frontier Science Center for Stem Cell Research, School of Life Sciences and Technology, Tongji University, Shanghai 200092, China; Frontier Science Center for Stem Cell Research, School of Life Sciences and Technology, Tongji University, Shanghai 200092, China; Frontier Science Center for Stem Cell Research, School of Life Sciences and Technology, Tongji University, Shanghai 200092, China; Frontier Science Center for Stem Cell Research, School of Life Sciences and Technology, Tongji University, Shanghai 200092, China; Frontier Science Center for Stem Cell Research, School of Life Sciences and Technology, Tongji University, Shanghai 200092, China; Key Laboratory of Spine and Spinal Cord Injury Repair and Regeneration of the Ministry of Education, Department of Orthopedics, Tongji Hospital, School of Life Sciences and Technology, Tongji University, Shanghai 200065, China; Frontier Science Center for Stem Cell Research, School of Life Sciences and Technology, Tongji University, Shanghai 200092, China; Key Laboratory of Spine and Spinal Cord Injury Repair and Regeneration of the Ministry of Education, Department of Orthopedics, Tongji Hospital, School of Life Sciences and Technology, Tongji University, Shanghai 200065, China; Frontier Science Center for Stem Cell Research, School of Life Sciences and Technology, Tongji University, Shanghai 200092, China; Shanghai Key Laboratory of Maternal Fetal Medicine, Shanghai Institute of Maternal-Fetal Medicine and Gynecologic Oncology, Clinical and Translation Research Center, Shanghai First Maternity and Infant Hospital, School of Life Sciences and Technology, Tongji University, Shanghai 200092, China; Frontier Science Center for Stem Cell Research, School of Life Sciences and Technology, Tongji University, Shanghai 200092, China; Key Laboratory of Spine and Spinal Cord Injury Repair and Regeneration of the Ministry of Education, Department of Orthopedics, Tongji Hospital, School of Life Sciences and Technology, Tongji University, Shanghai 200065, China; Frontier Science Center for Stem Cell Research, School of Life Sciences and Technology, Tongji University, Shanghai 200092, China; Shanghai Key Laboratory of Maternal Fetal Medicine, Shanghai Institute of Maternal-Fetal Medicine and Gynecologic Oncology, Clinical and Translation Research Center, Shanghai First Maternity and Infant Hospital, School of Life Sciences and Technology, Tongji University, Shanghai 200092, China; Frontier Science Center for Stem Cell Research, School of Life Sciences and Technology, Tongji University, Shanghai 200092, China; Key Laboratory of Spine and Spinal Cord Injury Repair and Regeneration of the Ministry of Education, Department of Orthopedics, Tongji Hospital, School of Life Sciences and Technology, Tongji University, Shanghai 200065, China; Frontier Science Center for Stem Cell Research, School of Life Sciences and Technology, Tongji University, Shanghai 200092, China

**Keywords:** gene regulation, transcription factor, TF footprint, chromatin landscape, chromatin accessibility, nucleosome position, chromatin remodeling

## Abstract

Gene regulation relies on the precise binding of transcription factors (TFs) at regulatory elements, but simultaneously detecting hundreds of TFs on chromatin is challenging. We developed cFOOT-seq, a cytosine deaminase-based TF footprinting assay, for high-resolution, quantitative genome-wide assessment of TF binding in both open and closed chromatin regions, even with small cell numbers. By utilizing the dsDNA deaminase SsdA_tox_, cFOOT-seq converts accessible cytosines to uracil while preserving genomic integrity, making it compatible with techniques like ATAC-seq for sensitive and cost-effective detection of TF occupancy at the single-molecule and single-cell level. Our approach enables the delineation of TF footprints, quantification of occupancy, and examination of chromatin influences on TF binding. Notably, cFOOT-seq, combined with FootTrack analysis, enables *de novo* prediction of TF binding sites and tracking of TF occupancy dynamics. We demonstrate its application in capturing cell type-specific TFs, analyzing TF dynamics during reprogramming, and revealing TF dependencies on chromatin remodelers. Overall, cFOOT-seq represents a robust approach for investigating the genome-wide dynamics of TF occupancy and elucidating the cis-regulatory architecture underlying gene regulation.

## Introduction

Chromatin accessibility, nucleosome arrangement, and transcription factors binding to cis-regulatory elements shape the genome’s regulatory landscape and dictate transcriptional activity (Kim and Wysocka, 2023; Klemm et al., 2019; Lambert et al., 2018). Mapping the genomic binding sites of all active transcription factors and their dynamic changes during cellular processes and environmental responses is crucial for understanding their roles in cell identity and the reshaping of gene regulatory networks (de Boer and Taipale, 2024; Gerstein et al., 2012; Spitz and Furlong, 2012; Vaquerizas et al., 2009). However, achieving this comprehensive mapping remains challenging due to the lack of sensitive and robust methods for large-scale dynamic assessment of genomic TF binding.

DNA binding specificities of TFs can be deduced by *in vitro* methods quantifying TF-DNA interaction by sequencing, such as SELEX (Jolma et al., 2010, 2013); however, it can’t provide the *in vivo* localization information of TFs. Occupancy of specific transcription factors on chromatin *in vivo* can be profiled by ChIP-seq (Gilmour and Lis, 1984; Johnson et al., 2007) or its optimized strategies such as ChIP-exo (He et al., 2015; Rhee and Pugh, 2011). Recently developed enzyme-tethering and cutting or tagging-dependent methods, including CUT&RUN (Skene and Henikoff, 2017), ChIL-seq (Harada et al., 2019), CUT&TAG (Kaya-Okur et al., 2019), ACT-seq (Carter et al., 2019), and CoBATCH (Wang et al., 2019), provide genome-wide distributions of chromatin-binding proteins with improved signal-to-noise ratios and lower sample requirements. Recent DNA-modifying enzyme-based approaches, such as DiMeLo-seq (Altemose et al., 2022), nanoHiMe-seq (Yue et al., 2022), and BIND&MODIFY (Weng et al., 2023), combine antibody-based protein recognition with protein A-fused nonspecific DNA adenine methyltransferases to map histone modifications and protein-DNA interactions, with methylation detection performed using PacBio or Nanopore technologies. While improvement on the throughput for single-cell and multiple targets (Ai et al., 2019; Bartosovic and Castelo-Branco, 2023; Bartosovic et al., 2021; Gopalan et al., 2021; Grosselin et al., 2019; Lochs et al., 2024; Meers et al., 2023; Xiong et al., 2024), the antibody-based strategies are still constrained by the necessity for highly specific antibodies and challenges in scalability, which make them hard to study the genomic kinetics of TF binding events for hundreds of TFs simultaneously.

TF binding can also be inferred from chromatin footprints caused by TF occupancy (Krebs, 2021; Sung et al., 2016). DNase-seq (He et al., 2014; Hesselberth et al., 2009; Neph et al., 2012a, 2012b) and ATAC-seq (Bentsen et al., 2020; Buenrostro et al., 2013; Hu et al., 2025; Li et al., 2019) have been used to detect the footprints of TF by identifying regions protected by TF from nuclease cleavage. Specifically, DNase-seq has been utilized in the ENCODE project to detect human TF footprints across hundreds of cell types (Vierstra et al., 2020). However, DNase-seq and ATAC-seq are dependent on numerous cutting events on open chromatin to robustly examine the protection effect from TF, which makes it hard to robustly detect TF occupancy on less accessible chromatin and in a lower number of cells, because of relative sparse cutting events. More recent advancements, including single-molecule footprinting (SMF) (Krebs et al., 2017; Sonmezer et al., 2021), SMAC-seq (Shipony et al., 2020), and Fiber-seq (Stergachis et al., 2020), employ DNA methyltransferases to modify accessible cytosines or adenines without DNA cleavage. However, SMF’s utility is limited by the sporadic occurrence of CpG and GpC sites, interference from endogenous cytosine methylation, and DNA degradation due to bisulfite conversion (Krebs, 2021). SMAC-seq and Fiber-seq address these issues by employing adenine methyltransferase and detecting DNA modifications via nanopore or PacBio sequencing (Shipony et al., 2020; Stergachis et al., 2020). Despite these improvements, third-generation sequencing methods are still hindered by accuracy, throughput, and cost, and require large cell quantities due to the inability to amplify modified DNA prior to sequencing.

Here, we present cFOOT-seq, a cytosine deaminase-based genomic footprinting assay by sequencing that simultaneously assesses chromatin accessibility, nucleosome positioning, and the occupancy of hundreds of transcription factors (TFs). cFOOT-seq leverages the dsDNA cytosine deaminase SsdA_tox_ to convert accessible cytosines to uracil, encoding chromatin organization directly into changes of DNA sequences. The positions of nucleosomes and TFs are inferred based on their protective effects against DNA deamination. cFOOT-seq is highly compatible with ATAC-seq, enabling cost-effective detection of TF occupancy in open chromatin and supporting detection of TF binding at the single-molecule and single-cell level. cFOOT-seq not only examines the dynamics of TF occupancy at known sites, but also provides *de novo* prediction of TF binding sites with FootTrack analysis of TF footprints and motifs. With cFOOT-seq, we analyzed the impact of chromatin context on TF occupancy and detected the dynamics of TF binding in the early stages of OSKM-mediated reprogramming of mouse embryonic fibroblasts (MEFs). We further defined the dependence of more than one hundred TFs on the SWI/SNF chromatin remodeling complex in HepG2, and revealed an observation of spatial organization that TFs with similar SWI/SNF dependency are frequently located in close proximity on the chromatin. We anticipate that future applications of cFOOT-seq will provide new insights into decoding the grammar of TF binding on chromatin and constructing accurate gene regulatory networks.

## Design

cFOOT-seq was developed to address the need for a sensitive and scalable method to map TF binding, chromatin accessibility, and nucleosome positioning (Klemm et al., 2019; Krebs, 2021). Traditional methods like ChIP-seq (Johnson et al., 2007) and CUT&Tag (Kaya-Okur et al., 2019) rely on antibodies, limiting scalability, while nuclease-based approaches like DNase-seq (Hesselberth et al., 2009) and ATAC-seq (Buenrostro et al., 2013) struggle with detecting TF binding in less accessible chromatin and require large cell inputs. Additionally, single-molecule and single-cell analyses remain challenging with these bulk-sequencing-dependent techniques (Preissl et al., 2022).

To address these limitations, cFOOT-seq leverages the dsDNA cytosine deaminase SsdA_tox_, which converts accessible cytosines to uracils, encoding chromatin structure and TF occupancy directly into DNA sequence changes. This design eliminates the need for nuclease cleavage or antibodies, preserving DNA integrity while enabling the detection of TF footprints in both open and closed chromatin regions. cFOOT-seq is highly adaptable and can be integrated with complementary approaches, such as ATAC-seq, to enrich for open chromatin regions. This integration enhances detection sensitivity and reduces sequencing costs. Notably, the combined ATAC-cFOOT workflow extends the utility of cFOOT-seq to single-molecule and single-cell level, enabling detailed mapping of TF binding at unprecedented resolution. cFOOT-seq can examine the dynamics of TF occupancy at known sites, and provides *de novo* prediction of TF binding sites with FootTrack analysis. By addressing limitations related to cell number, chromatin openness, and enzyme biases, cFOOT-seq provides a more accurate and comprehensive understanding of TF dynamics and chromatin architecture across the genome.

## Results

### cFOOT-seq maps chromatin accessibility, nucleosome positioning, and TF footprints

**Figure 1.**
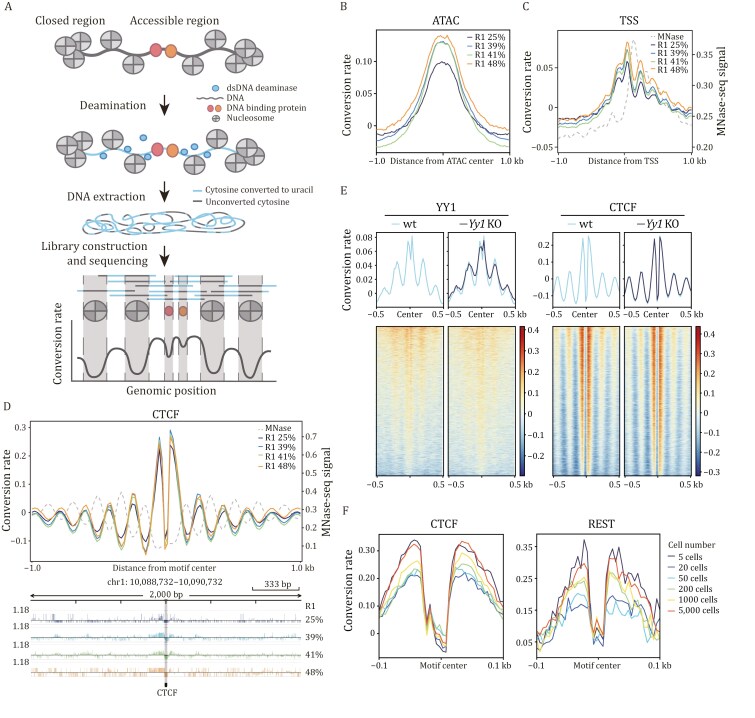
cFOOT-seq maps chromatin accessibility, nucleosome occupancy, and TF footprints. (A) Schematic of the cFOOT-seq workflow. After cell permeabilization, dsDNA deaminases convert cytosine to uracil in accessible chromatin DNA, but DNA occupied by nucleosomes or transcription factors (TFs) remains unconverted. The profile of DNA conversion rate defines nucleosome occupancy and TF footprints. (B) Average profile of normalized DNA conversion rate around the centers of open chromatin regions (OCR), which are defined by ATAC-seq peaks in R1 cells, showing samples with increasing average genomic conversion rate. (C) Average profile of normalized DNA conversion rate of cFOOT-seq and MNase-seq signal around transcription start sites (TSS) in R1 cells, showing samples treated with increasing average genomic conversion rate. The pattern of nucleosome positioning, as demonstrated by MNase-seq, is depicted by the gray dashed line. (D) Average profiles of normalized DNA conversion rate of cFOOT-seq and MNase-seq signal around CTCF binding sites defined by ChIP-seq in R1 cells, displaying the nucleosome position and CTCF footprint. The pattern of nucleosome positioning as indicated by MNase-seq is shown with a gray dashed line (top). Integrative Genomic Viewer (IGV) browser view of a representative site (chr1: 10,088,732–10,090,732) flanking the CTCF motif showing the distribution of DNA conversion rate around CTCF motifs at single site (bottom). The position of CTCF motif is marked by gray bar. (E) Average profiles of normalized DNA conversion rate around YY1 (left) and CTCF (right) binding motifs in wild-type (wt, 33%) and *Yy1* knockout (KO, 32%) R1 cells, displaying the changes of YY1 footprint after knockout of *Yy1*. Heatmaps showing DNA conversion rate around individual binding sites aligned to motif centers and ranked by average conversion rate within ±0.1 kb of the motif center (bottom). The binding sites of YY1 and CTCF are defined by ChIP-seq in R1. (F) Average profiles of normalized DNA conversion rate around CTCF (left) or REST (right) binding motifs in R1 samples with low input cell numbers (5–5,000), displaying the footprints of CTCF and REST at their known binding sites. The binding sites of CTCF and REST are defined by ChIP-seq in R1. See also Figs. S1 and S2.

To convert chromatin architecture into DNA sequences at single-nucleotide resolution, we developed cFOOT-seq, a cytosine deaminase-based genomic footprinting assay by sequencing that depicts chromatin accessibility, nucleosome positioning, and transcription factor occupancy. In cFOOT-seq, permeabilized cells are treated with double-stranded DNA (dsDNA) cytosine deaminases, which preferentially convert accessible cytosine (C) to uracil (U). PCR amplification subsequently results in C-to-T conversions. Open chromatin, being more susceptible to deamination, shows a higher conversion rate, indicating higher chromatin accessibility. Conversely, nucleosome and TF binding protect the DNA from deamination, revealing their footprints as regions with decreased conversion rate (Fig. 1A).

For mapping the chromatin landscape and TF footprints across the genome, DNA deaminases with robust enzymatic activity and minimal sequence bias on dsDNA are essential. While DddA_tox_ was initially identified for its ability to deaminate cytosine in dsDNA, its TC context preference limits its use for TF footprint detection (Mok et al., 2020). SsdA_tox_ (de Moraes et al., 2021), along with recently identified DddA_tox_ homologs like Ddd_Ss (Mi et al., 2023), offer more potent enzymatic activity and reduced sequence bias. To evaluate their activity and sequence bias for cFOOT-seq, we purified SsdA_tox_, Ddd_Ss, and another DddA_tox_ homolog, Ddd_Fa, for comparison (Fig. S1A).

Our findings demonstrate that SsdA_tox_ exhibits more consistent deamination across a range of DNA oligonucleotides compared to Ddd_Ss and Ddd_Fa (Fig. S1B), with significantly lower sequence bias. In tests using naked genomic DNA from R1 cells, SsdA_tox_ shows rapid deamination, achieving near 100% conversion at a relatively low enzyme concentration, while Ddd_Ss and Ddd_Fa exhibit a more gradual increase in conversion rate with increasing enzyme concentration (Fig. S1C and S1D). This highlights SsdA_tox_’s superior catalytic efficiency compared to Ddd_Ss and Ddd_Fa.

Nucleotide preference studies reveal that Ddd_Fa retains a strong TC context bias, while Ddd_Ss exhibits less sequence specificity (Fig. S1E and S1F). In contrast, SsdA_tox_ shows minimal sequence bias, making it ideal for comprehensive genomic analysis (Fig. S1F). When assessing CTCF motifs on R1 naked genomic DNA, SsdA_tox_ demonstrated consistent performance with minimal sequence bias, whereas Ddd_Ss and Ddd_Fa showed greater variability, reinforcing SsdA_tox_’s suitability for precise TF footprint detection (Fig. S1G).

Moreover, SsdA_tox_ is less affected by DNA methylation status compared to Ddd_Ss and Ddd_Fa, as evidenced by its deamination efficiency at both high and low methylation sites (Fig. S1H). Mass spectrometry analysis of deamination at C and 5mC sites further supports that SsdA_tox_ deaminates 5mC more efficiently than the other enzymes (Fig. S1I). This characteristic is crucial for detecting TF footprints in DNA regions with prevalent methylation. With its high activity and minimal bias, SsdA_tox_ is the optimal choice for use in cFOOT-seq, ensuring accurate representation of chromatin organization across broad sequence coverage.

Treating permeabilized R1 cells with increasing SsdA_tox_ concentrations, we observed a higher genomic conversion rate correlating with higher SsdA_tox_ concentrations (Fig. S2A). Conversion rate at ATAC-seq peaks and transcription start sites (TSS) were higher than those in flanking regions (Fig. 1B and 1C), suggesting that these conversion rate reflect chromatin accessibility. This pattern was consistent across multiple cell types, including human HepG2 cells, further demonstrating the robustness of the assay (Fig. S2B and S2C).

cFOOT-seq detected nucleosome phasing patterns around TSS and CTCF binding sites, consistent with the nucleosome positioning signal from MNase-seq (Fig. 1C and 1D). Conversion rate at TSS revealed downstream nucleosome positioning patterns (Fig. 1C). The conversion rate around CTCF motif also showed strong nucleosome phasing patterns, consistent across samples with different conversion rate after normalization (Fig. 1D). Similar nucleosome phasing patterns were observed around the binding sites of REST and YY1 (Fig. S2D and S2E), indicating a unique chromatin landscape around their binding sites.

cFOOT-seq was able to detect TF occupancy sites based on their footprints. Conversion rate at the CTCF motif was significantly lower than that around the motifs, representing CTCF footprint at binding sites due to protection from deamination (Fig. 1A and 1D). Footprints were also detected at REST and YY1 binding sites (Fig. S2D and S2E). Knocking out *Yy1* in R1 cells led to a notable reduction in both nucleosome phasing and footprint signals around YY1-binding motifs, while signals around CTCF motifs were unaffected, confirming YY1 occupancy signals detected by cFOOT-seq are specific to YY1 (Figs. 1E and S2F). Moreover, the loss of YY1 footprints can also be observed at individual binding sites in *Yy1* KO R1 cells (Fig. S2G), further supporting the specificity of the detected footprint signals.

cFOOT-seq captures both nucleosome positions and TF footprints through deamination, rather than cutting chromatin DNA, providing a more comprehensive view of chromatin status in both open chromatin regions (OCR) and closed chromatin regions (CCR). Compared to DNase-seq and ATAC-seq, cFOOT-seq successfully detects CTCF footprints in both OCR and CCR, revealing nucleosome phasing around the CTCF binding sites (Fig. S2H). While ATAC-seq and DNase-seq can detect CTCF footprints in OCR, their ability to identify footprints in CCR is limited, and they lack detailed chromatin context, such as nucleosome phasing. Similarly, cFOOT-seq detects TF CEBPA footprints in both chromatin contexts (Fig. S2H), further highlighting its advantage in simultaneously capturing chromatin structure and TF occupancy across the genome over DNase-seq and ATAC-seq.

Finally, we assessed cFOOT-seq’s ability to characterize chromatin landscapes using limited cell numbers. Remarkably, the assay could detect chromatin accessibility, nucleosome phasing patterns around CTCF, and footprints of CTCF and REST in as few as 5–5,000 R1 cells (Figs. 1F, S2I, and S2J). In all, cFOOT-seq provides high-resolution insights into chromatin primary structure, encompassing chromatin accessibility, nucleosome positioning, and TF occupancy, even with minimal cell numbers, making it a powerful tool for studying chromatin dynamics and TF binding across diverse cell types and conditions.

### cFOOT-seq quantitatively measures TF occupancy

To comprehensively assess transcription factor occupancy and dynamics under various conditions through TF footprint analysis, we developed an analysis framework named FootTrack (footprint analysis for tracking TF occupancy and kinetics) (Fig. S3A), which conceptionally adapted from TOBIAS and footprint-tools (Bentsen et al., 2020; Vierstra et al., 2020). Using known TF binding information and motif information, FootTrack precisely maps TF occupancy and the chromatin landscape around TF motif centers at known binding sites. By integrating cFOOT-seq data and motif information from JASPAR (Rauluseviciute et al., 2024), FootTrack also facilitates *de novo* prediction of transcription factor binding sites genome-wide.

**Figure 2.**
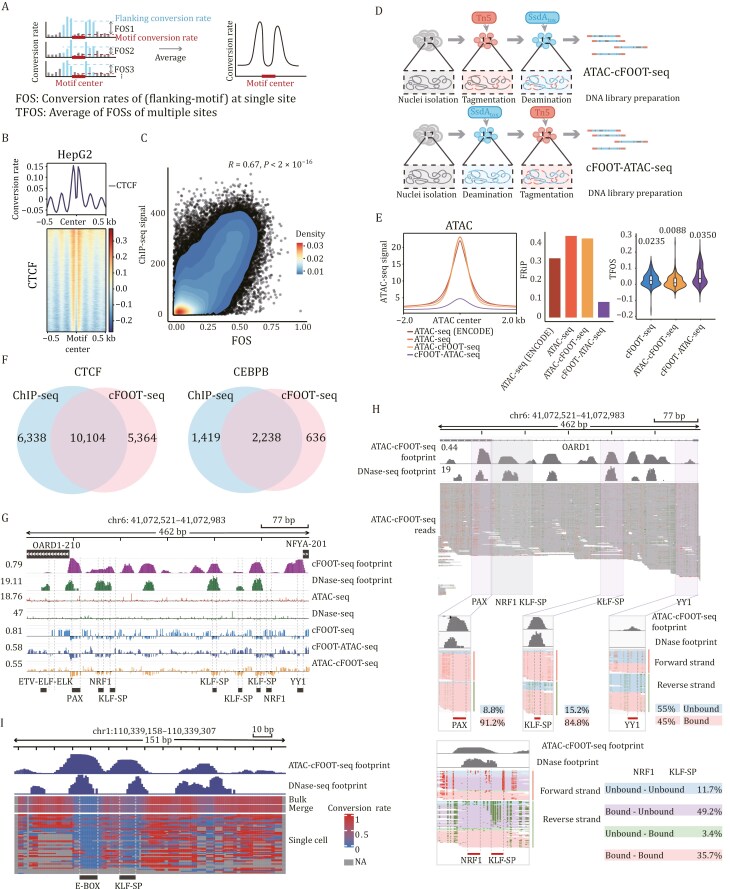
cFOOT-seq combined with ATAC-seq detects TF footprints at single-molecule and single-cell level. (A) Schematic of calculations for footprint occupancy score (FOS) and transcription factor occupancy score (TFOS). FOS is defined as the difference between the average conversion rate in the 50 bp flanking regions on both side of the motif and the average conversion rate of the motif itself, representing the occupancy information at individual binding motif. TFOS is calculated as the average FOS across multiple binding sites of the same TF, reflecting the overall TF occupancy. (B) Average profile of corrected DNA conversion rate around CTCF binding sites defined by ChIP-seq in HepG2 cells (30%). Heatmaps showing DNA conversion rate around individual CTCF binding sites aligned to motif centers and ranked by average conversion rate within ±0.5 kb of the motif center (bottom) around CTCF binding sites. (C) The scatter plot illustrates the Pearson correlation between cFOOT-seq and ChIP-seq signals at CTCF binding sites. cFOOT-seq signals are quantified using the footprint occupancy score (FOS), including only sites with a FOS greater than 0. The ChIP-seq signal is calculated as the average value around each motif, extending ±50 bp from the motif’s center. The correlation coefficient is 0.67, with a highly significant *P*-value of less than 2 × 10^−16^. The color gradient indicates the density of data points. (D) Workflow of cFOOT-ATAC-seq and ATAC-cFOOT-seq methods: cFOOT-ATAC-seq involves SsdA_tox_ deaminase incubation followed by Tn5-mediated tagmentation (top); ATAC-cFOOT-seq involves Tn5-mediated tagmentation followed by SsdA_tox_ deaminase incubation (bottom). (E) Comparison of ATAC-seq, cFOOT-seq (30%), ATAC-cFOOT-seq (33%), and cFOOT-ATAC-seq (36%) methods: read depth around ±2 kb of the ATAC peak center based on 10 million reads, using two ATAC-seq datasets (one from ENCODE and one generated in-house) as benchmarks for evaluating open chromatin enrichment (left), fraction of reads in peaks (FRiP) (middle), and distribution of TFOS (right). (F) Venn diagrams showing the overlap of CTCF and CEBPB footprints predicted by FootTrack-based *de novo* analysis of cFOOT-seq and CTCF binding motifs observed by ChIP-seq peaks in open chromatin regions of HepG2. (G) IGV browser graphics showing profiles of cFOOT-seq, cFOOT-ATAC-seq, ATAC-cFOOT-seq, DNase-seq, and ATAC-seq at a representative site (chr6: 41,072,521–41,072,983) in HepG2 cells. The “cFOOT footprint” track shows footprint scores derived from cFOOT-seq data, while the “DNase footprint” track shows footprint probabilities from DNase-seq data. The five tracks below display the distribution of corrected cutting events for ATAC-seq and DNase-seq, along with the corrected conversion rate for cFOOT-seq, cFOOT-ATAC-seq, and ATAC-cFOOT-seq. The black labels at the bottom highlight motifs identified in ChIP-seq peaks, corresponding to TF binding sites. (H) IGV browser visualization showing the single-molecule profile of ATAC-cFOOT-seq at a representative site (chr6: 41,072,521–41,072,983) in HepG2 cells. The “ATAC-cFOOT-seq footprint” track represents footprint scores calculated from ATAC-cFOOT-seq data, while the “DNase footprint” track shows footprint probabilities predicted from DNase-seq data. The “ATAC-cFOOT-seq reads” track displays the individual mapped reads in this region. Both forward and reverse strands are shown for the three TF motifs (PAX, KLF-SP, and YY1), with reads categorized into bound and unbound states based on conversion rate at each motif. The bottom panel visualizes the distribution of reads for two adjacent motifs (NRF1 and KLF-SP), categorized into four groups based on the presence or absence of TF binding, providing insight into their single-molecule occupancy patterns. (I) IGV browser visualization showing scATAC-cFOOT-seq profiles at a representative site (chr1: 110,339,158–110,339,307) in K562 cells (38%). The “ATAC-cFOOT-seq footprint” track shows footprint scores calculated from bulk ATAC-cFOOT-seq data, while the “DNase footprint” track represents footprint probabilities from DNase-seq data. Below, conversion rate profiles for bulk (top), merged (middle), and single-cell (bottom) data are displayed, with the conversion rate color scale ranging from 0 to 1. A 5-bp smoothing window is applied to the single-cell data, and sites with no data are marked in gray. The motifs E-BOX and KLF-SP, predicted in the footprint regions, are shown below the plot. See also Figs. S3, S4, S5, and Tables S1 and S2.

We quantified TF occupancy using the footprint occupancy score (FOS), calculated as the difference between the average DNA conversion rate in the 50 bp flanking regions on either side of the motif and the motif center (Fig. 2A), similar to FOS calculation in DNase-seq (Neph et al., 2012b). Averaging FOS across all binding sites or specific regions yields the transcription factor occupancy score (TFOS), which reflects general TF occupancy.

To optimize cFOOT-seq procedures and FootTrack analysis parameters, we selected HepG2 and K562 cell lines due to the extensive TF binding data available for these model cells. To assess potential biases in cFOOT-seq’s detection of TF footprints due to the sequence context preference of SsdA_tox_, we analyzed the footprint of the HepG2-specific factor HNF1B in K562 cells. We observed that HNF1B also exhibited clear footprints in K562, suggesting that SsdA_tox_’s sequence bias could distort quantitative assessments of TF occupancy (Fig. S3B). To address this, we treated naked genomic DNA with SsdA_tox_ and calculated the sequence context bias probability in human and mouse genomes (Fig. S3C). After correcting for this bias, the distortion in TF footprints was significantly reduced (Fig. S3B and S3C).

We further optimized the enzyme concentrations for cFOOT-seq. We selected 204 TFs with known localization and motif information in HepG2 cells (Table S1) for optimization testing and further analysis (Moyers et al., 2023; Partridge et al., 2020). HepG2 cells were treated with varying enzyme units, revealing that at conversion rate of 25% and 30%, both TFOS and the percentage of sites with positive FOS for each TF were similar (Fig. S3D). However, when the conversion rate exceeded 40%, both TFOS and the percentage of sites with positive FOS decreased. Therefore, we determined that a conversion rate range of 25%–40% is optimal for accurately measuring most TFs. Next, we assessed the required sequencing depth for accurate FOS measurement. We found that a minimum of approximately 15 million reads (~1.4× depth) per whole-genome sequencing sample is necessary for TFOS assessment, with approximately 200 million paired reads (~6.6× depth) providing good stability and 400–800 million reads offering even higher reliability (Fig. S3E).

With these optimizations, FootTrack accurately depicted nucleosome organization around CTCF binding sites and measured CTCF occupancy in HepG2 cells using cFOOT-seq data (Fig. 2B). By calculating the FOS for CTCF binding motifs, we observed strong correlations between FOS and ChIP-seq signals (Fig. 2C), suggesting that FOS can serve as a reliable quantitative measure of transcription factor occupancy.

### cFOOT-seq combined with ATAC-seq provides high-resolution and sensitive detection of TF footprint

Compared to DNase-seq and ATAC-seq, cFOOT-seq converts chromatin structure information into DNA sequence data without disrupting chromatin structure, allowing combination with other chromatin-probing technologies. We explored combining cFOOT-seq with ATAC-seq to enrich accessible chromatin and measure TF occupancy at a lower sequencing cost. We tested two combined strategies based on the order of treatment of SsdA_tox_ and Tn5: (i) ATAC-cFOOT-seq (ATAC followed by cFOOT-seq, Tn5 fragmentation followed by SsdA_tox_ deamination) and (ii) cFOOT-ATAC-seq (cFOOT followed by ATAC-seq, SsdA_tox_ deamination followed by Tn5 fragmentation) in HepG2 and K562 cells (Fig. 2D).

ATAC-cFOOT-seq efficiently enriches open regions similar to ATAC-seq and detects footprints for most TFs, with TFOS generally comparable to those identified by cFOOT-seq in HepG2 (Figs. 2E, S4A, and S4B). However, for certain TFs such as FOXA1, their TFOS were reduced (Fig. S4B), likely due to TF binding loss during the ATAC-cFOOT-seq process. cFOOT-ATAC-seq also demonstrates obvious enrichment of open regions compared to cFOOT-seq (Fig. S4C), though weaker than ATAC-cFOOT-seq (Fig. 2E). Importantly, cFOOT-ATAC-seq detected higher TFOS than cFOOT-seq, suggesting its greater stability and sensitivity in detecting TF binding (Figs. 2E, S4A, and S4B). We noticed that the fraction of reads in peaks (FRiP) of cFOOT-ATAC-seq is negatively correlated with the conversion efficiency (Fig. S4D). When conversion efficiency is between 20% and 30%, cFOOT-ATAC-seq achieves higher enrichment of open regions while maintaining sensitive TF binding detection in K562 (Fig. S4D and S4E). In summary, ATAC-cFOOT-seq and cFOOT-ATAC-seq each offer distinct advantages in detecting TF footprints and enriching open chromatin regions, making them powerful and complementary techniques for TF footprint analysis.

DNase-seq has long been a benchmark for TF footprint detection (He et al., 2014; Hesselberth et al., 2009; Neph et al., 2012b; Vierstra et al., 2020), and ATAC-seq, with optimized bioinformatics is becoming increasingly popular (Bentsen et al., 2020; Hu et al., 2025; Li et al., 2019). By analyzing metrics including FWHM and Integral Width, we compared cFOOT-seq and its combined methods to DNase-seq and ATAC-seq for footprint resolution of 204 TFs in HepG2. While DNase-seq outperforms ATAC-seq in some instances, our results show that cFOOT-seq and its combined methods, particularly cFOOT-ATAC-seq, offer higher resolution overall (Fig. S4F).

Using TFs such as ATF4, CEBPD, HNF1A, and FOXA1, we further evaluated the resolution and sensitivity of these methods at different sequencing depths. At 13.2 M and 1.65 M reads, ATAC-seq struggles to detect footprints, underscoring its dependence on sequencing depth (Fig. S4G). In contrast, cFOOT-seq and DNase-seq perform well, with cFOOT-seq detecting sharper footprints for ATF4, CEBPD, and HNF1A (Fig. S4G). Notably, while DNase-seq struggles with FOXA1, cFOOT-seq reliably detects its footprints. At extremely lower sequencing depths (0.33 M reads), both DNase-seq and cFOOT-seq show decreased stability in footprint detection (Fig. S4G). However, the combined methods (ATAC-cFOOT-seq and cFOOT-ATAC-seq) maintain robust detection for all four TFs (Fig. S4G). This demonstrates the advantage of combining cFOOT-seq with ATAC-seq, which not only enriches open chromatin regions but also utilizes deaminase-mediated DNA conversion for more reliable and sensitive footprint detection, even at reduced sequencing depths.

Next, we used FootTrack to *de novo* identify genome-wide TF footprints from the DNA conversion data generated by cFOOT-seq. To avoid interference from nucleosome occupancy, FootTrack was specifically applied to open chromatin regions across the genome, enabling the analysis of TF binding dynamics and occupancy changes. FootTrack offers two background correction modes: global background and local background. Following the background correction, FootTrack proposed two different strategies for predicting footprints: S1, calculates footprint scores for each motif with bias corrected data, and applies statistical methods to identify motifs with higher scores as potential TF binding sites, and S2, initially detects footprint regions by binomial statistical test and then scans for motifs within the identified footprint regions (Fig. S3A). Based on the performance evaluation, we found that local mode performs better than global mode for both S1 and S2, and S1 performed better for cFOOT-seq, while S2 performs better for ATAC-cFOOT-seq and cFOOT-ATAC-seq (Fig. S4H).

To validate the accuracy of footprint prediction, we examined representative TFs with well-characterized ChIP-seq binding profiles. The footprints of CTCF and CEBPB identified by FootTrack in open chromatin regions showed strong overlap with motifs located within ChIP-seq peaks in HepG2 (Fig. 2F). We further applied FootTrack to cFOOT-seq and cFOOT-ATAC-seq data from HepG2, K562, and R1 cells to quantify footprint density in open chromatin regions (OCR). Footprint density was similar across cell types (~4.2 footprints per 200 bp; Fig. S4I–L). Notably, enhancer-associated OCR consistently showed higher footprint density than promoter-associated OCR, suggesting more extensive TF binding at enhancers (Vierstra et al., 2020). To assess how many footprints could be linked to known TFs, we compared predicted footprints in open regions with known motifs. About 60% matched known motifs, while ~40% lacked recognizable matches (Fig. S4M–O). Although footprint scores were similar, footprints without known motifs were generally broader, potentially reflecting binding by larger chromatin-associated proteins, TF complexes, or cooperative TF interactions. Notably, an increasing number of TF pairs have been found to form composite motifs distinct from their individual canonical motifs (Jolma et al., 2015; Xie et al., 2025). Since our analysis primarily relied on the JASPAR database, incorporating additional motif sources and accounting for composite motif architectures in future work may further improve the annotation of currently unassigned footprints.

### cFOOT-seq combined with ATAC-seq enables detection of TF footprint at single-molecular and single-cell level

Accurate detection of TF occupancy at single loci is critical for footprint-based methods to elucidate transcription factor function. Using cFOOT-seq and its combined methods, we analyzed two regions (chr6: 41,072,521–41,072,983 and chr1: 110,338,780–110,339,310) and found that cFOOT-seq and its combined methods predict footprints that overlap with those defined by DNase-seq and motifs in ChIP-seq peaks, demonstrating their reliability in detecting TF footprints at these sites in a *de novo* manner (Figs. 2G and S5A).

Combining cFOOT-seq with ATAC-seq enriches open chromatin regions, enabling higher sequencing depth and more precise quantification of TF occupancy at both single-molecule and single-cell levels. At the single-molecule level, TF occupancy is quantified by analyzing conversion rate at footprint sites, using each read covering the corresponding motif. For example, at region 1 (chr6: 41,072,521–41,072,983), variations in TF occupancy across different motifs are observed: PAX motif (ATAC-cFOOT-seq, 91.2%; cFOOT-ATAC-seq, 98.3%), KLF-SP motif (ATAC-cFOOT-seq, 84.8%; cFOOT-ATAC-seq, 76.9%), and YY1 motif (ATAC-cFOOT-seq, 45%; cFOOT-ATAC-seq, 69.4%) (Figs. 2H and S5B). Similar analyses for the two E-box motifs and ZBTB33 at region 2 further confirm this capability (Fig. S5C and S5D). These findings underscore cFOOT-seq’s ability to capture detailed TF occupancy insights through conversion patterns in footprint reads. Additionally, single-molecule conversion profiles allow the analysis of TF occupancy at nearby sites, such as NRF1 and KLF-SP motifs in region 1 (Fig. 2H). Notably, 49.2% of cases show NRF1 bound and KLF-SP unbound, 35.7% show both NRF1 and KLF-SP bound together, and only 3.4% show KLF-SP bound without NRF1, suggesting a potential dependence of KLF-SP binding on NRF1 at this site. This ability to detect TF co-occupancy within the same read forms the basis for studying synergistic binding events between TFs.

By integrating plate-based single-cell library preparation technology, we enable TF footprint detection at the single-cell level through scATAC-cFOOT-seq (single-cell ATAC combined with cFOOT-seq). After Tn5 tagmentation and SsdA_tox_ deamination, cells are sorted into individual wells of a 96-well plate. Following cell lysis, DNA is barcoded by preamplification using well-specific primers, and the samples are pooled for further library amplification (Fig. S5E). A total of 71 barcodes (Table S2) were used to label 71 K562 cells, and 100 million reads were sequenced (~1.3 million reads per cell). The scATAC-cFOOT-seq assay yields a median FRiP of approximately 0.4, comparable to that of other well-established methods (Fig. S5F). TF footprint signals at the binding sites of CTCF and NRF1 were observed in individual cells (Fig. S5G). Notably, TF footprints can even be detected at single genomic loci within individual cells (Fig. 2I), demonstrating the ability to analyze TF occupancy in heterogeneous cell populations. Together, cFOOT-seq combined with ATAC-seq provides a high-resolution, powerful tool for detecting TF footprints at both the single-molecule and single-cell levels, offering valuable insights into gene regulatory networks, TF interactions, and chromatin dynamics.

### cFOOT-seq quantitatively assess the impact of chromatin accessibility, histone modifications, and cofactors on TF occupancy

TF binding is influenced by chromatin accessibility and histone modifications, but large-scale systematic analysis is limited. Now cFOOT-seq provides us a chance to quantitatively analyze factors associated with TF binding.

To investigate the impact of chromatin accessibility on TF occupancy, we analyzed 204 TFs in HepG2 and 127 TFs in K562 with known binding sites and motifs (Table S1). We categorized their binding sites into four groups: closed chromatin regions (CCR), low, moderate, and high open chromatin regions (OCR). Our analysis revealed a general trend where higher chromatin accessibility correlated with increased TFOS (Fig. S6A and S6B). While many TFs displayed higher TFOS in highly accessible regions, some TFs with lower overall TFOS showed no significant change across different levels of chromatin accessibility (Fig. S6A).

**Figure 3.**
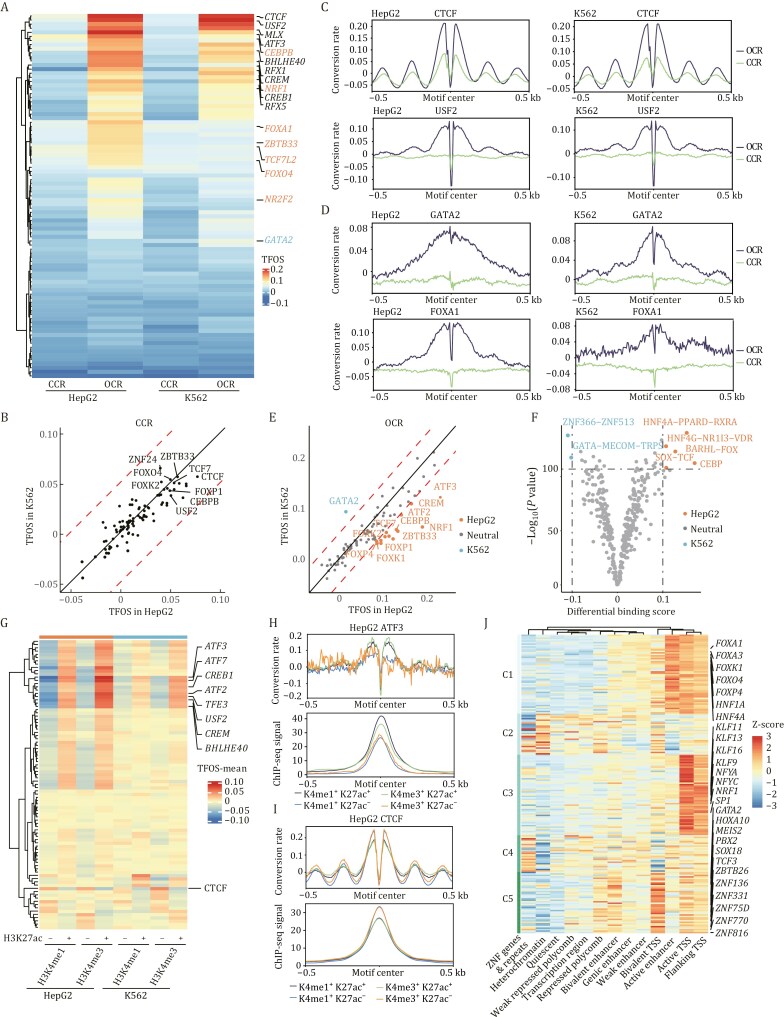
cFOOT-seq quantitatively assess the impact of chromatin accessibility, histone modifications, and cofactors on TF occupancy. (A) Heatmap of TFOS at closed chromatin regions (CCR) and open chromatin regions (OCR) of 89 overlapping TFs with known binding sites and motifs in HepG2 (30%) and K562 (30%) cells. CCR and OCR are defined by ATAC-seq in each cell type. TFs with higher TFOS in OCR of HepG2 or K562 are indicated in the figure as described in the legend. The intensity scale reflects TFOS, with values ranging from low to high. (B) Scatter plot comparing the TFOS in CCR between HepG2 and K562 cells. Each point represents an individual transcription factor. TFs with TFOS (in CCR) >0.05 in both cells are highlighted. The red dashed lines denote a threshold where the difference in TFOS between the two cell types exceeds 0.04. (C and D) Average profiles of corrected DNA conversion rate around the binding sites of CTCF and USF2 (C), GATA2 and FOXA1 (D) in HepG2 and K562 cells, highlighting their differences of TFOS in CCR and OCR, and TFOS difference between cell lines. (E) Scatter plot comparing the TFOS of TFs at known binding sites in OCR between HepG2 and K562 cells. The dashed lines denote a threshold where the difference in TFOS between the two cell types exceeds 0.04. TFs with TFOS values greater by more than 0.04 in either HepG2 or K562 are labeled accordingly in the figure. (F) Volcano plot showing the TF clusters with differential binding scores between K562 and HepG2 cells, based on *de novo* binding sites predicted by FootTrack. TFs with significantly higher binding scores in K562 or HepG2 are shown on the left and right sides of the plot, respectively, as labeled in the figure. (G) Heatmap showing TFOS in regions with different histone modification status (H3K4me1^+^H3K27ac^−^, H3K4me1^+^H3K27ac^+^, H3K4me3^+^H3K27ac^−^, and H3K4me3^+^H3K27ac^+^) of 89 overlapping TFs in HepG2 and K562 cells. (H and I) Average profiles showing corrected DNA conversion rate (top) and ChIP-seq signal (bottom) around ATF3 (H) and CTCF (I) binding sites in HepG2 cells. Binding sites were defined by ChIP-seq of each TF. Both cFOOT-seq and ChIP-seq signals are shown separately for regions marked by different combinations of histone modifications (H3K4me1^+^H3K27ac^−^, H3K4me1^+^H3K27ac^+^, H3K4me3^+^H3K27ac^−^, and H3K4me3^+^H3K27ac^+^) of HepG2 cells. (J) Heatmap showing TFOS Z-score in regions defined by chromHMM annotations for 204 TFs in HepG2 cells. The TFs were clustered into five groups, with the representative TF highlighted. See also Fig. S6, and Tables S1 and S3.

We further selected 89 overlapping TFs from both cell lines and clustered them based on their TFOS in CCR and OCR (Fig. 3A). The differences in TFOS between closed and open regions were generally consistent between HepG2 and K562 (Fig. S6C), indicating that chromatin accessibility similarly influences TF occupancy in both cell types. Among the 89 TFs analyzed, CTCF, ZNF24, ZBTB33, TCF7, USF2, CEBPB, and FOX family proteins exhibited high TFOS (>0.05) in CCR in both cell types (Fig. 3B). This indicates these proteins have a stronger ability to bind closed chromatin. Consistently, FOX proteins have been proposed as pioneer factors capable of accessing closed chromatin. Additionally, CTCF, bHLH E-box binding factors (USF2, TFE3, MLX, and BHLHE40), bZIP factors (CREB1, CREM, and ATF3/7), and RFX family proteins (RFX1/5) demonstrated high TFOS in OCR in both cell lines, with TFOS in open regions being significantly higher than in closed regions (Figs. 3A, 3C, and S6A). This suggests their preferential binding to accessible chromatin.

Comparing TFOS between HepG2 and K562 cells revealed potential cell-specific transcription factors. While TFOS in CCR was generally similar between the two cell types, notable differences were observed in OCR (Fig. 3B, 3D, and 3E). HepG2 cells exhibited higher TFOS for factors such as NRF1, NR2F2, TCF7L2, ZBTB33, and bZIP family members (ATF3, CEBPB/CEBPG, and CREM), and FOX family proteins, whereas K562 cells showed higher TFOS for GATA2 (Fig. 3D and 3E). To further identify cell-specific factors, we utilized FootTrack for *de novo* footprint analysis. This analysis predicted higher scores for clusters such as CEBP, BARHL–FOX, HNF4A–PPARD–RXRA, SOX–TCF, in HepG2, and for the GATA cluster in K562 (Fig. 3F). These predictions were consistent with the cFOOT-seq signal at known TF binding sites derived from ChIP-seq (Figs. 3E and S6D), demonstrating the predictive capability of FootTrack.

To investigate how co-binding influences TF occupancy, we used TF-COMB (Bentsen et al., 2022) to identify co-occurring TF pairs in open chromatin regions based on ChIP-seq data for 204 TFs in HepG2, 127 in K562, and 27 in R1. Top-ranked pairs such as ZBED4–EGR1 (HepG2, Fig. S6E) and SP1–MAZ (K562, Fig. S6F) showed enhanced TFOS in shared regions compared to individual binding sites, and both TF pairs showed higher chromatin accessibility in co-bound regions, suggesting that TF co-occupancy may promote chromatin accessibility and enhance TF binding. Further Motif spacing analysis revealed ZBED4–EGR1 displayed a broader spacing distribution, suggesting less spatial constraint and flexible chromatin co-binding (Fig. S6E), while SP1–MAZ showed a strong bias toward near-zero spacing (Fig. S6F), likely due to motif similarity and overlapping binding, which may cause steric hindrance. Thap11 and ZNF143 were identified as a prominent co-binding pair in R1 cells. Their motif centers were enriched at an 8 bp spacing, consistent with previous reports of composite motif formation (Vinckevicius et al., 2015) (Fig. S6G). Notably, co-occupancy markedly increased ZNF143’s TFOS, while Thap11 was only modestly affected (Fig. S6G), indicating that ZNF143 may depend on Thap11 for stable chromatin association. Together, these findings suggest that cooperative binding and increased chromatin accessibility are key contributors to enhanced TFOS in shared regions, while steric hindrance may also play a role in specific TF combinations.

To investigate the effect of histone modifications on TF occupancy, we analyzed the TFOS of 89 TFs in HepG2 and K562 across four chromatin states: H3K4me1^+^H3K27ac^−^, H3K4me1^+^H3K27ac^+^, H3K4me3^+^H3K27ac^−^, and H3K4me3^+^H3K27ac^+^ (Fig. 3G). Generally, TFOS was higher in H3K27ac^+^ regions compared to H3K27ac^−^ regions. Specifically, bHLH E-box binding factors (USF2, TFE3, and BHLHE40) and bZIP factors (CREB1, CREM, and ATF2/3/7) exhibited significantly higher TFOS in H3K27ac^+^ regions than in H3K27ac^−^ regions, regardless of H3K4me1 or H3K4me3 presence, consistent with the trends observed in ChIP-seq data (Figs. 3G, 3H, and S6H). Moreover, the TFOS of CTCF was notably lower in H3K27ac^+^ regions compared to H3K27ac^−^ regions (Figs. 3I and S6I). This trend was also evident in ChIP-seq data, suggesting a negative correlation between CTCF binding and the presence of H3K27ac or its associated proteins.

To comprehensively analyze the impact of chromatin context on TF occupancy, we clustered 204 TFs in HepG2 cells into five groups based on their TFOS in regions defined by chromHMM annotations (Ernst and Kellis, 2012) (Fig. 3J; Table S3). We found that cluster 1, enriched with liver-specific factors and FOX family proteins, had the highest TFOS in active enhancer regions, consistent with their role as tissue-specific pioneer factors that open chromatin at enhancers to regulate liver-specific genes. Cluster 3 was enriched with promoter-binding factors, which had the highest TFOS at transcription start sites (TSS) and TSS-flanking regions. Cluster 5 included TFs involved in embryonic development and cell fate determination, as well as ZNF proteins, which had the highest TFOS in bivalent TSS regions. This indicates that TFs tend to have higher binding affinity in their preferred regions, where they play key functional roles, suggesting significant binding specificity and functional specialization of TFs. The specialization of TFs to distinct genomic contexts underscores the intricate nature of TF-chromatin interactions and their critical role in shaping the regulatory landscape of the genome.

### cFOOT-seq could capture the dynamics of TF in early stage of OSKM reprogramming

Unraveling core transcription factor regulatory networks is crucial for understanding cell identity during differentiation and reprogramming (Neph et al., 2012a). Using cFOOT-seq, we analyzed TF binding differences between MEF and R1 cells. By inputting the open chromatin regions of MEF and R1 into FootTrack, we identified TFs enriched in each cell type (Fig. S7A). Further analysis with TF-COMB revealed that AP family TFs form a closely co-occurrence network in MEF, while stemness-related factors such as SOX family proteins and SALL4 form a network in R1 (Fig. S7B). These results suggest that cFOOT-seq effectively captures cell type-specific TFs and provides insights into TF co-occurrence network.

**Figure 4.**
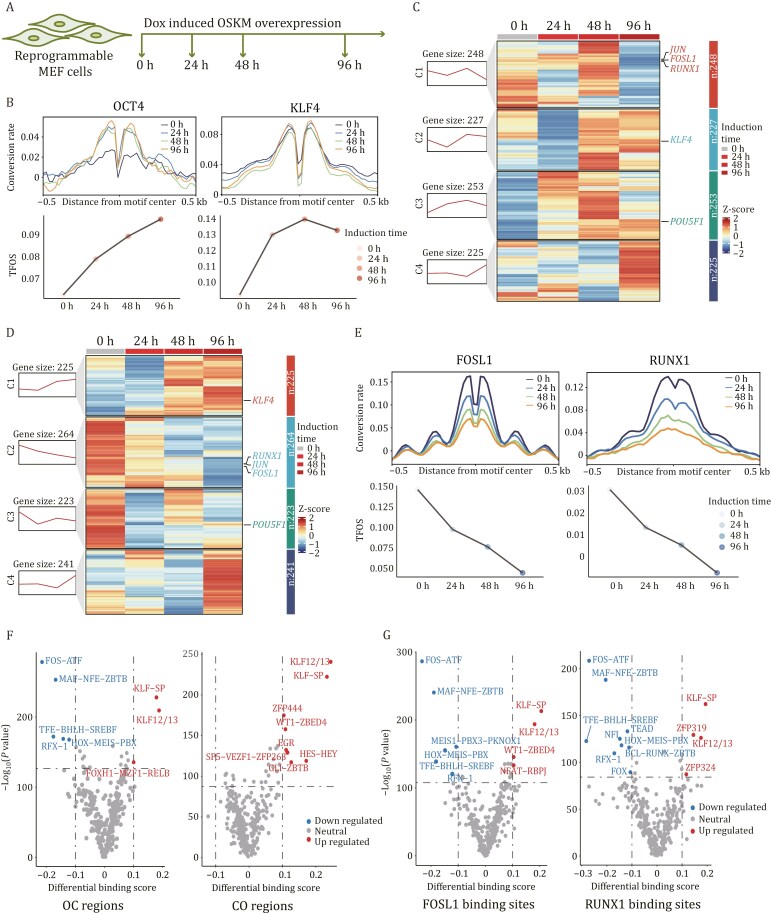
cFOOT-seq could capture the dynamics of TFs in early stage of OSKM reprogramming. (A) Schematic of experimental treatments on reprogrammable MEF cells with doxycycline-induced OSKM overexpression. (B) Average profiles and box plots showing changes in OCT4 (left) and KLF4 (right) footprints over induction times (0 h-34%, 24 h-34%, 48 h-35%, and 96 h-37%) in OSKM-MEF cells. Average profiles display corrected DNA conversion rate aligned at OCT4 and KLF4 binding sites in R1. Line plots show TFOS changes representing dynamics of TF occupancy. (C) Heatmaps of FootTrack-predicted TF footprints in R1-specific open regions at different induction times (0 h, 24 h, 48 h, and 96 h), clustered into four groups by footprint changes. (D) Heatmaps of FootTrack-predicted TF footprints in MEF-specific open regions at different induction times (0 h, 24 h, 48 h, 96 h), clustered into four groups by footprint changes. (E) Average profiles and box plots showing changes in FOSL1 and RUNX1 footprints over time in OSKM-MEF cells. Average profiles display corrected DNA conversion rate aligned at FOSL1 and RUNX1 known binding sites defined by ChIP-seq in R1. Line plots show TFOS changes representing dynamics of TF occupancy. (F) Volcano plots showing the FootTrack-predicted TF clusters with differential binding scores between 0 h and 48 h in OSKM-MEF cells for OC regions (open in MEF, closed in R1) and CO regions (closed in MEF, open in R1). TFs with significantly decreased or increased footprint scores at 48 h are categorized as downregulated or upregulated, respectively, as indicated in the figure. (G) Volcano plots showing the FootTrack-predicted TF clusters with differential binding scores between 0 h and 48 h in OSKM-MEF cells for FOSL1 and RUNX1 binding regions in MEF cells. TFs with significantly decreased or increased footprint scores at 48 h are categorized as downregulated or upregulated, respectively, as indicated in the figure. See also Fig. S7.

The reprogramming of MEF to iPSC is coupled with dramatic changes of chromatin accessibility and TFs binding (Chronis et al., 2017; Li et al., 2017). To assess if cFOOT-seq could capture TF dynamics during reprogramming, we used an inducible OSKM system to reprogram MEF to iPS cells. Doxycycline (Dox) was added for 24 h, 48 h, and 96 h to induce OSKM expression, and TF footprints were detected using cFOOT-seq (Fig. 4A). TFOS of OCT4 and KLF4 increased rapidly after 24 h of induction (Fig. 4B). Conversion rate at the flanking sites of the OCT4 binding motif increased after Dox addition, while the rate at the flanking sites of the KLF4 motif remained high and unchanged (Fig. 4B). This suggests that OCT4 acts as a pioneer factor opening chromatin at closed sites, whereas KLF4 binds to pre-accessible chromatin in MEF post-induction.

To systematically analyze TF changes, we used FootTrack to predict TF occupancy dynamics in MEF-specific and R1-specific open regions during early reprogramming, clustering TFs based on TFOS variation trends (Fig. 4C and 4D). TFOS for JUN, FOSL1, and RUNX1 decreased significantly in MEF-specific regions (Fig. 4C), while KLF4 and POU5F1 increased in both MEF and R1-specific open regions (Fig. 4D). The TFOS decrease of FOSL1 and RUNX1 was confirmed by the cFOOT-seq signal at known TF binding sites defined by ChIP-seq (Fig. 4E), supporting that the binding of these proteins is decreased during the reprogramming of MEF to R1. TF-COMB analysis has revealed that AP family TF interactions are more specific to MEF (Fig. S7B), the reduced AP family binding is required for MEF reprogramming towards iPS cells, consistent with the idea that AP family proteins act as reprogramming barriers (Li et al., 2017; Liu et al., 2021).

Based on differential chromatin accessibility between MEF and R1, we defined OC regions as those open in MEF but closed in R1, and CO regions as those closed in MEF but open in R1. We further analyzed the potential driving forces behind changes in chromatin accessibility during reprogramming and found that KLF family proteins increased in both OC and CO regions (Fig. 4F). These results suggest that increased binding of KLF proteins may be correlated with changes of chromatin accessibility during reprogramming. Additionally, further analysis of the binding sites of FOSL1 and RUNX1 in OC regions indicated that KLF proteins might be associated with the suppression of FOSL1 and RUNX1 (Fig. 4G) (Liu et al., 2021), highlighting their role in gene regulation during the reprogramming process.

### cFOOT-seq depicts dynamics of nucleosome organization and TF occupancy in response to inhibition of SWI/SNF

Chromatin remodeling complexes regulate gene expression by modulating the organization of nucleosome through ATP-dependent translocase activity (Becker and Workman, 2013; Bracken et al., 2019; Clapier et al., 2017; Eustermann et al., 2024). SWI/SNF remodeler could open chromatin by nucleosome sliding and eviction, facilitate the binding of TFs, and its mutations or dysfunction are linked to various cancers and developmental disorders (Ahmad et al., 2024; Cenik and Shilatifard, 2021; Kadoch et al., 2013; Shain and Pollack, 2013). Although there have been efforts to explore the effect of SWI/SNF complex on chromatin accessibility and the binding of TFs (Alver et al., 2017; Barisic et al., 2019; Brahma and Henikoff, 2024; Iurlaro et al., 2021; Martin et al., 2023; Schick et al., 2021), a comprehensive understanding of TF dependence on SWI/SNF remains limited due to the lack of high-throughput methods for accurately and quantitatively measuring TF occupancy on chromatin. To address this, we used cFOOT-seq to study chromatin accessibility around TFs and dynamics of TF occupancy following SWI/SNF inhibition with compound BRM014 in mESC cells and HepG2 cells (Papillon et al., 2018).

In mESC treated with BRM014 for 1 h and 24 h, we observed a general decrease in conversion rate around ATAC-seq peaks, indicating reduced chromatin accessibility (Fig. S8A and S8B). Consistent with previous reports, TFs such as OCT4 and REST showed significant decreases in DNA conversion rate around the binding sites of OCT4 and REST, along with a reduction in TFOS for these factors, while CTCF binding sites remained unchanged (Fig. S8C) (Iurlaro et al., 2021). FootTrack predicts that TF clusters, including GLI-ZIC, TFE-BHLH-SREBF, bHLH-PAS, ESRR-NR, KLF, and MYC proteins, had decreased footprint scores (Fig. S8D). Published ChIP-seq data confirmed that the footprints of MYCN and KLF4 decreased after BRM014 treatment (Fig. S8E). However, the chromatin accessibility pattern around the binding sites of MYCN and KLF4 largely remained unchanged (Fig. S8E).

**Figure 5.**
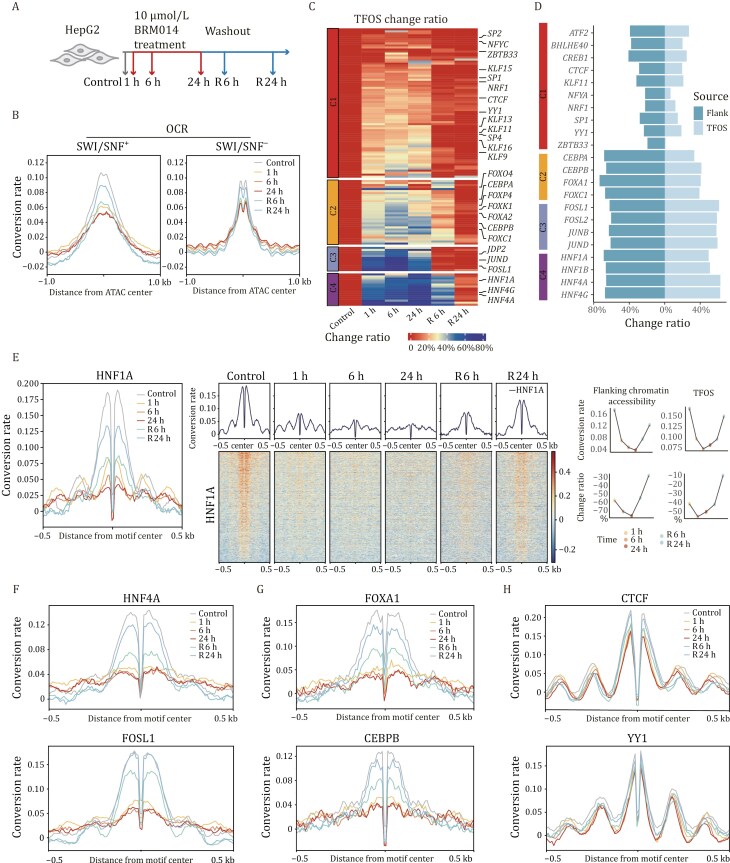
cFOOT-seq depicts dynamics of nucleosome organization and TF occupancy in response to inhibition of SWI/SNF. (A) Schematic representation of the experimental design depicting the treatment of HepG2 cells with 10 μmol/L BRM014 for 1 h, 6 h, and 24 h, as well as 6 h and 24 h recovery after 24 h BRM014 treatment. (B) Average profiles of DNA conversion rate around OCR with or without SWI/SNF binding sites defined by ChIP-seq, illustrating changes in chromatin accessibility following different durations of BRM014 treatment and recovery (Control 0 h-32%, 1 h-31%, 6 h-32%, 24 h-29%, R6 h-33%, R24 h-33%). (C) Heatmap showing TFOS change ratio in SWI/SNF^+^ regions across different BRM014 treatment conditions for 134 filtered TFs in HepG2 cells. The TFs are clustered into four groups based on change ratio of TFOS across treatments. Grid color represents the dynamic change ratio compared with controls. (D) Butterfly graph shows flanking chromatin accessibility change ratio (left) and TFOS change ratio (right) of representative TFs in different clusters, which represent changes in flanking accessibility and TF occupancy. (E) Average profiles and heatmap of overall DNA conversion rate around HNF1A binding sites in SWI/SNF^+^ regions (left), illustrating changes in chromatin accessibility, nucleosome position and TF occupancy after BRM014 treatment and recovery. Plot graphs of flanking chromatin accessibility, TFOS and their change ratio are shown (right). Flanking chromatin accessibility around the TF binding motifs are calculated as the average DNA conversion rate in the 50 bp flanking regions on both sides of the motif as flanking chromatin accessibility. (F–H) Average profiles of DNA conversion rate around TF binding sites of HNF4A and FOSL1 (F), FOXA1 and CEBPB (G), CTCF and YY1 (H) under control, 1 h, 6 h, and 24 h BRM014 treatments, as well as 6 h and 24 h recovery after 24 h BRM014 treatment. The profiles show changes in chromatin accessibility and TF footprints across different treatment conditions. See also Fig. S8 and Table S4.

To comprehensively analyze the effect of SWI/SNF inhibition on nucleosome organization and TF occupancy, we selected HepG2 cells as our model system due to the extensive TF binding data available from the ENCODE project. HepG2 cells were treated with the SWI/SNF inhibitor BRM014 for 1 h, 6 h, and 24 h, followed by recovery periods of 6 h and 24 h after the 24 h treatment (Fig. 5A). Similar to the observations in R1 cells, the conversion rate around ATAC-seq peaks in HepG2 decreased rapidly in response to BRM014 treatment and recovered upon inhibitor removal (Fig. S8F). ATAC-seq peaks overlapping with the SWI/SNF complex showed a more dramatic decrease compared to those without SWI/SNF, consistent with the inhibitory effect of BRM014 on the SWI/SNF complex (Fig. 5B).

To accurately assess changes in TF occupancy, we calculated the TFOS at SWI/SNF^+^ sites for individual TFs and determined the TFOS change ratio post-treatment. To ensure reliability, we filtered out TFs with unrobust TFOS as mentioned in methods, resulting in a selection of 134 qualified TFs. We then profiled their dynamic responses to BRM014 treatment by clustering TFs based on their TFOS change ratios (Fig. 5C; Table S4). To measure chromatin accessibility flanking the TF binding sites, we calculated the average DNA conversion rate in the 50 bp flanking regions on both sides of the motif as flanking chromatin accessibility. Based on these clusters, we further calculated the change ratio of flanking chromatin accessibility post-treatment (Fig. S8G). Representative TFs in clusters 3 and 4 showed a significant decrease in both flanking chromatin accessibility and TFOS following treatment, with TFs in cluster 3 recovering more quickly than those in cluster 4 (Figs. 5C, 5D, and S8G). TFs in cluster 2 exhibited a marked decrease in flanking chromatin accessibility but only a mild reduction in TFOS. Conversely, TFs in cluster 1 demonstrated minimal decreases in both flanking chromatin accessibility and TFOS (Figs. 5C, 5D, and S8G).

We further analyzed the conversion rate kinetics around TF binding sites of representative TFs to assess dynamics of flanking chromatin accessibility and nucleosome organization. For cluster 4, HNF1A at SWI/SNF^+^ sites showed a dramatic decrease in TFOS and conversion rate of flanking chromatin around its binding sites after 1 h of BRM014 treatment, with nucleosome positions moving closer to the binding sites (Fig. 5E). This suggests a quick loss of HNF1A binding, chromatin accessibility and nucleosome repositioning. After 6 h and 24 h of treatment, the nucleosome phasing pattern further diminished, likely due to the loss of the anchoring effect caused by HNF1A binding. Similar to HNF1A, decrease of flaking chromatin accessibility and TF binding were observed for HNF1B/4A/4G in cluster 4, and AP-1 family proteins (FOSL1/2, JUNB/D) in cluster 3 which are highly sensitive to BRM014, suggesting the chromatin accessibility around the binding motifs and TF occupancy of these proteins are largely dependent on SWI/SNF (Figs. 5E, 5F, and S8H).

For cluster 2, the conversion rate around the binding sites of the FOX family is also decreased dramatically, suggesting the chromatin accessibility around their binding sites are dependent on SWI/SNF. However, compared to HNF proteins and AP-1 family proteins, the TFOS change ratios of FOX family proteins are lower (Fig. 5D and 5G), indicating that their binding on chromatin is less dependent on the opening of the chromatin by SWI/SNF, which is also consistent with the pioneer activity of FOX proteins to bind closed chromatin.

For cluster 1, CTCF showed only mild decreases in TFOS and chromatin accessibility around their binding sites (Fig. 5H). Consistent with this observation, NURF-specific subunit BPTF and ISWI core translocase SNF2H has been reported to maintain the organization of nucleosomes and local accessibility around CTCF sites (Bomber et al., 2023; Iurlaro et al., 2024; Wiechens et al., 2016). Additionally, NRF1, NFY complex, YY1, and ZBTB33 were resistant to BRM014 treatment (Figs. 5H and S8I). It is highly possible that like CTCF, these proteins may also dependent on other remodeling factors to open chromatin, which need further investigation (Cai et al., 2007; Iurlaro et al., 2024).

### Definition of TF dependence on SWI/SNF reveals the spatial organization rule of TFs

**Figure 6.**
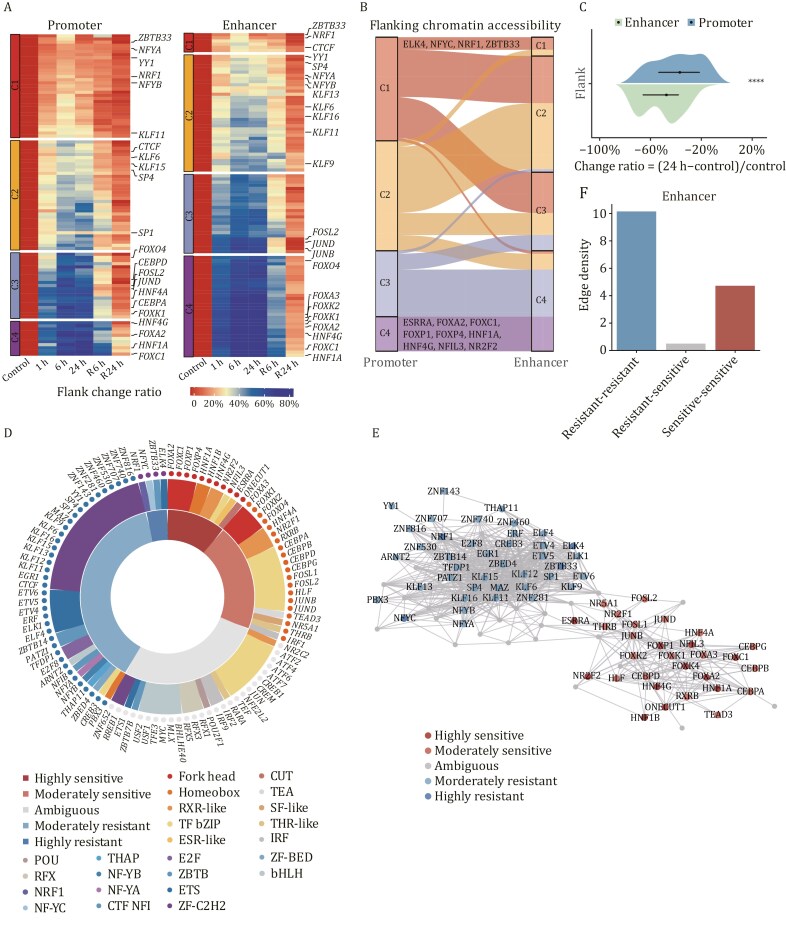
Definition of TF dependence on SWI/SNF reveals the spatial organization rule of TFs. (A) Heatmap showing the change ratio of flanking chromatin accessibility around TF binding sites at promoter (left) and enhancer (right) for 100 TFs in HegG2 cells. TFs are grouped into four clusters based on their change ratios at different time points following BRM014 treatment. Grid color represents the dynamic change ratio compared with controls. (B) Sankey diagram illustrating the correspondence between the enhancer and promoter clustering categories for each TF as defined in panel (A). TFs highlighted in cluster 1 or cluster 4 in both promoter and enhancer regions represent those that are highly resistant or highly sensitive to BRM014 treatment. (C) Violin plot showing the distribution of change ratios in flanking chromatin accessibility for enhancers and promoters in HepG2 cells after 24 h of BRM014 treatment, with statistical significance indicated (****P value < 0.0001). (D) Sunburst plot illustrating the sensitivity of TFs to BRM014 treatment, organized by clusters and families in HepG2 cells. The inner circle categorizes TFs into highly sensitive, moderately sensitive, ambiguous, moderately resistant, and highly resistant groups. The middle circle indicates TF family classifications. Individual TFs are labeled in the outer ring. All category and family groupings are visually labeled in the figure. (E) The network diagram displays the transcription factor co-occurrence network with the enhancer regions of HepG2. Each node represents a different TF, and edges between nodes indicate potential co-occurrence relationships among the TFs. TFs are categorized by their sensitivity or resistance to BRM014 treatment, with groupings indicated in the figure. (F) The bar graph displays the edge density in transcription factor co-occurrence network within the enhancer regions of HepG2 cells among transcription factor pairs, categorized by their sensitivity to BRM014. The categories include interactions between TF pairs that are both resistant, one resistant and one sensitive, and both sensitive as labeled in the figure. See also Fig. S9 and Table S5.

Previous reports have shown that chromatin accessibility at promoters and enhancers may respond differently to the deletion or inhibition of SWI/SNF (Alver et al., 2017; Basurto-Cayuela et al., 2024; Martin et al., 2023). We also found that compared to promoters, the overall conversion rate of enhancers decreased more obviously after BRM014 treatment in HepG2, suggesting that the chromatin accessibility of enhancers is more dependent on SWI/SNF complex (Fig. S9A). To evaluate the differential effect of enhancers and promoters on TF dependence of SWI/SNF, we separated the TF binding sites by promoter and enhancer, and calculated their flanking chromatin accessibility. To ensure reliability of analysis, we further filtered out TFs with unrobust score in promoter and enhancer as mentioned in methods, resulting in a selection of 100 qualified TFs from 134 TFs. Using consensus clustering, we separated the TFs into four clusters based on their change ratio of flanking chromatin accessibility on promoter or enhancer (Table S5). From clusters 1 to cluster 4, they represent the TFs, whose flanking chromatin accessibility in enhancer or promoter are highly resistant, moderately resistant, moderately sensitive and highly sensitive to BRM014 treatment (Fig. 6A). We found that some TFs has similar sensitivity to BRM014 at both enhancer and promoter; however, some TFs display different sensitivity to BRM014 between at enhancer and promoter. Generally, flanking chromatin accessibility of TFs in enhancers is decreased more dramatically than in promoter, indicating that the sensitivity of TFs to BRM014 can be chromatin context-dependent (Fig. 6B and 6C).

We defined the sensitive TF and resistant TF based on their sensitivity consistency of flanking chromatin accessibility on promoter and enhancer. The TFs are classified to highly resistant TF (C1 in both promoter and enhancer), moderately resistant TF (either C1 or C2 in both promoter and enhancer), moderately sensitive TF (either C3 or C4 in both promoter and enhancer), and highly sensitive TF (C4 in both promoter and enhancer) (Fig. 6B). Resistant TFs enrich CTCF, NRF1, NFYA/B/C, YY1, ZBTB33, SP1/4, KLF proteins, ETS proteins, and ZNF proteins, while sensitive TFs enrich HNF tissue-specific factors, RXR-like family, FOX family, AP-1 family, and C/EBP family (Fig. 6D). TFs within the same family recognizing similar motifs shared comparable SWI/SNF dependency, highlighting evolutionary conservation in SWI/SNF dependency (Fig. 6D).

To characterize the functional differences of TFs in each cluster, we evaluated each TF’s regulatory preference by calculating the proportion of its binding sites overlapping promoter regions (±1 kb) of HepG2-specific versus housekeeping genes. Violin plot analysis showed that SWI/SNF-sensitive TFs are more likely to target cell-type-specific genes, whereas insensitive TFs preferentially bind to housekeeping gene promoters (Fig. S9B, left). GO enrichment analysis of the HepG2-specific gene set revealed that the top-enriched terms were related to small-molecule catabolism and biosynthesis, consistent with the known metabolic functions of liver cells (Fig. S9B, right). Together, these results suggest that SWI/SNF-sensitive TFs are associated with specialized regulatory programs, while insensitive TFs are involved in fundamental cellular processes.

By integrating SWI/SNF dependency information, we further analyzed TF organizational patterns in enhancers and promoters in HepG2 cells by TF-COMB. Interestingly, TFs with the same SWI/SNF dependency prefer to locate closer to each other, irrespective of at enhancer and promoter (Figs. 6E and S9C), with co-occurrence of TFs within the same group significantly more than TFs between different dependency groups (Figs. 6F and S9D). Given that chromatin remodelers reshape chromatin by consuming ATP, we hypothesize that this spatial organization ensures both stability and plasticity of chromatin organization during genome regulation of development and signaling response in an energy-efficient manner. Further characterization of TF organization in other cells is crucial to determine if this spatial organization is conserved and its significance in gene regulation.

## Discussion

Genomic-wide capture of TF binding dynamics on chromatin is essential for unraveling gene regulatory networks and understanding cellular transcriptional changes during development and in response to external signals (de Boer and Taipale, 2024; Kim and Wysocka, 2023). We developed cFOOT-seq, which employs dsDNA deaminase to encode chromatin structure information into genomic DNA sequences. This approach enables simultaneous profiling of chromatin accessibility, nucleosome positioning, and TF occupancy on a genome-wide scale. cFOOT-seq measures TF occupancy based on TF footprints in both open and closed chromatin, capturing the dynamics of hundreds of TFs in a single experiment. We developed the FootTrack analysis framework to trace the dynamics of TF occupancy based on either known binding sites or *de novo* predictions. By leveraging known TF binding information, FootTrack can accurately and quantitatively track the dynamics of hundreds of TFs in HepG2 and K562 cells. In addition, FootTrack can *de novo* predict TF occupancy from footprints and motifs, enabling the identification of potential TF candidates involved in transcriptional regulation. As a proof of concept, cFOOT-seq successfully detected TF dynamics during OKSM-mediated reprogramming, profiled TF dependencies on SWI/SNF, and uncovered a spatial organization rule where TFs with the same SWI/SNF dependence localize closely in HepG2. Thus, cFOOT-seq provides a novel genomics toolkit for studying gene regulatory networks and chromatin organization.

By encoding chromatin structure into DNA sequences, cFOOT-seq preserves DNA integrity, allowing for single-molecule and single-cell TF occupancy detection (Fig. 2H and 2I). When combined with ATAC-seq, cFOOT-seq enriches open chromatin regions, improving TF binding detection at lower costs. The increased sequencing depth in open chromatin allows more sensitive and quantitative TF footprint detection at the single-molecule level, enabling accurate quantification of TF binding and opening new opportunities for analyzing TF cooperation.

Integrating plate-based single-cell technology with ATAC-cFOOT-seq (Figs. S5E–G and 2I) enables high-resolution TF occupancy profiling in heterogeneous populations, providing a powerful tool for mapping gene regulatory networks. With increased throughput, scATAC-cFOOT-seq could be widely applied to uncover the regulatory principles underlying cell fate specification, developmental dynamics, and disease progression. Moreover, the compatibility of cFOOT-seq with long-read sequencing platforms, such as PacBio and Nanopore, offers unique opportunities to analyze repetitive genomic regions and cooperative interactions between cis-regulatory elements.

cFOOT-seq and its combination with ATAC-seq provide complementary advantages. cFOOT-seq uniquely profiles both genome-wide chromatin accessibility and TF footprints, though reliable footprint detection typically requires ~200 million reads per sample. ATAC-cFOOT-seq, similar to the recently published FOODie method (He et al., 2024), uses Tn5-mediated open chromatin enrichment before deamination, enabling sensitive footprint detection with lower sequencing depth but potentially missing footprints due to Tn5 incubation process (Fig. 2E). In contrast, cFOOT-ATAC-seq, while offering lower open chromatin enrichment, yet achieves more sensitive TF footprint detection (Fig. 2E). It is important to note that combined methods relying on Tn5 enrichment no longer yield conversion rates that accurately reflect true genome-wide accessibility. By preserving this quantitative relationship, cFOOT-seq enables parallel analysis of TF binding patterns and intrinsic chromatin openness, which is particularly valuable for studying regulatory dynamics across different chromatin states (Fig. 5).

Traditionally, genome-wide mapping of regulatory factor binding sites relied on antibody-based methods like ChIP-seq and newer techniques like CUT&RUN and CUT&TAG, which detect one protein at a time. In contrast, footprint-based methods can simultaneously address hundreds of TFs in one experiment, identifying occupied sites with nucleotide precision. cFOOT-seq data offers comprehensive, high-resolution insights into the spatial and temporal dynamics of TF binding, elucidating regulatory networks and gene expression mechanisms in various biological processes.

### Limitations

Despite its strengths, cFOOT-seq has certain limitations, including residual enzyme sequence bias, and challenges in transcription factor (TF) assignment. Unbiased enzymatic activity is essential for accurate TF footprint detection. Among the tested deaminases, SsdA^tox^ exhibits the lowest sequence bias compared to DddA, Ddd_Fa, and Ddd_Ss (Mi et al., 2023), making it particularly suitable for high-precision footprint profiling. DddB, recently applied in the FOODie method, displays a weaker bias than DddA but still obviously prefers TC over GC motif (He et al., 2024). A systematic comparison of SsdA^tox^ and DddB will be important for selecting the most suitable deaminases in future footprinting studies. Current correction approaches largely minimize the residual bias of SsdA^tox^, yet some motif-specific effects remain detectable. Further improvements, such as deep learning-based correction models (Eraslan et al., 2019; Hu et al., 2025), may enhance analytical accuracy. Additionally, engineering SsdA^tox^ variants with reduced sequence bias could further improve the method.

The limited availability of known motifs for certain TFs restricts the scope of cFOOT-seq, and incorporating additional motif databases could broaden TF coverage. Moreover, performing *de novo* motif discovery based on FootTrack-predicted footprints could further expand its applicability. Accurate footprint detection for AT-rich motifs is more challenging due to the lack of cytosines, underscoring the need for complementary approaches. Footprint assignment is further complicated by motif redundancy among TF families, which can be partially addressed using expression data and validated through ChIP-seq or related methods. Expanding the motif repertoire, including novel and composite motifs, together with a deeper understanding of TF binding grammar, will enhance the accuracy and applicability of cFOOT-seq.

## Supplementary Material

pwaf071_Supplementary_Materials

## Data Availability

Data related to motifs were sourced from JASPAR (Rauluseviciute et al., 2024) while ChIP-seq and ATAC-seq datasets were obtained from Cistrome (Zheng et al., 2019) and ENCODE. The specific data sources can be found in Table S1. The cFOOT-seq sequencing data generated in this study (Table S8) have been archived and are accessible at the Sequence Read Archive (SRA; BioProject accession number PRJNA1140376). Further information and requests for reagents may be directed to the Lead Contact, Jia-Min Zhang (zhangjiamin@tongji.edu.cn).

## Code availability

The code for FootTrack analysis is available at github. com/ZhangLab-TJ/FootTrack.

## Artificial intelligence (AI)

We used AI-assisted technologies (ChatGPT, OpenAI) solely for language polishing and grammar improvement of the manuscript. All scientific content, analyses, and conclusions were conceived, written, and verified by the authors.
