# Supplementary material for: Genome-wide investigation of transcription factor footprints and dynamics using cFOOT-seq
Source: Protein Cell. 2025 Aug 4;16(11):932–52. doi: 10.1093/procel/pwaf071 (PMC12698189; doi:10.1093/procel/pwaf071)
Supplement: pwaf071_Supplementary_Materials [file pwaf071_supplementary_materials.zip › pwaf071_suppl_Supplementary_Methods_for_cFOOT-seq.docx]

**Methods**

**Key resources table**

| REAGENT or RESOURCE | SOURCE | IDENTIFIER |
| --- | --- | --- |
| Antibodies | | |
| YY1 | Cell Signaling | Cat# 46395, RRID:AB_279930 |
| GAPDH | Proteintech | Cat# CL488-60004, RRID:AB_2919223 |
| Bacterial and virus strains | | |
| Rosetta™ 2 (DE3) Singles™ Competent Cells | EMD Millipore | Cat# 71400-4 |
| Chemicals, peptides, and recombinant proteins | | |
| MEM with NEAA | Procell | Cat# PM150410 |
| RPMI 1640 | Thermo Fisher Scientific | Cat# 11875093 |
| DMEM-high glucose | Life Technologies | Cat# C11960500BT |
| FBS | Gibco | Cat# 10270106 |
| L-glutamine | Thermo Fisher Scientific | Cat# 25030164 |
| 100× nucleosides | EMD Millipore | Cat# ES-008-D |
| 100× NEAA | Millipore | Cat# TMS-001 |
| 2-mercaptoethanol | Sigma-Aldrich | Cat# M6250 |
| Mouse LIF Protein | Millipore | Cat# ESG1107 |
| 100× penicillin/streptomycin | Gibco | Cat# 15140122 |
| PD0325901 MEK inhibitor | Selleck | Cat# S1036 |
| CHIR99021 GSK3 inhibitor | Selleck | Cat# S1263 |
| IPTG | Sigma-Aldrich | Cat# I6758-1G |
| BRM/BRG1 ATP Inhibitor-1 | MedChem Express | Cat# HY-119374 |
| Uracil-DNA Glycosylase (UDG) | NEB | Cat# M0280S |
| TaKaRa Taq™ Hot Start Version | TaKaRa | Cat# R007 |
| nuclease P1 | NEB | Cat# M0660 |
| Quick CIP | NEB | Cat# M0525 |
| protease K | Qiagen | Cat# 19157 |
| Tn5 | Vazyme | Cat# S601 |
| SsdAtox | This paper | N/A |
| Ddd_Ss | This paper | N/A |
| Ddd_Fa | This paper | N/A |
| Critical commercial assays | | |
| EpiArt DNA Methylation Library Kit | Vazyme | Cat# NE-103 |
| EndoFree Plasmid Maxi Kit | CoWin Biotech Co. | Cat# 029332 |
| SanPrep Column DNA Gel Extraction Kit | Sangon | Cat# B518131 |
| Deposited data | | |
| Raw and analyzed data | This paper | Reviewer link： https://dataview.ncbi.nlm.nih.gov/object/PRJNA1140376?reviewer=err25fb3vgman253umfb4vg5so  https://ngdc.cncb.ac.cn/gsa/s/JK7nM9fI |
| See Table S1 for accession number of TF ChIP-seq, ATAC-seq and other data of different cell line | This paper | Table S1 |
| Experimental models: Cell lines | | |
| HepG2 | Procell | Cat# CL-0103, RRID：CVCL_0027 |
| K562 | Procell | Cat# CL-0130, RRID：CVCL_0004 |
| R1 | ATCC | Cat# SCRC-1011, RRID:CVCL_2167 |
| mESC-OG2 | Wang et al.(Wang et al., 2022) | N/A |
| Reprogrammable MEF | Xi et al.(Xi et al., 2022) | N/A |
| Oligonucleotides | | |
| See Table S6 for oligo sequence | This paper | Table S6 |
| Recombinant DNA | | |
| lentiGuide vector | Addgene | Cat# 52963, RRID: Addgene_139449 |
| pETduet-SsdAtox-SsdAI | This paper | N/A |
| pCOLADuet1-10His-Ddd_Fa | Mi et al. (Mi et al., 2023) | N/A |
| pCOLADuet1-10His-Ddd_Ss | Mi et al. (Mi et al., 2023) | N/A |
| Software and algorithms | | |
| FastQC (v0.12.1) | N/A | https://www.bioinformatics.babraham.ac.uk/projects/fastqc |
| Trim Galore (v0.6.10) | N/A | <https://www.bioinformatics.babraham.ac.uk/projects/trim_galore> |
| BASAL (v1.3) | Xu. et al. (Xu et al., 2025) | <https://github.com/JiejunShi/BASAL> |
| deepTools | Ramírez et al. (Ramirez et al., 2016) | https://deeptools.readthedocs.io/en/latest/ |
| TOBIAS (v0.16.1) | Bentsen et al. (Bentsen et al., 2020) | https://github.com/loosolab/TOBIAS |
| FootTrack | This paper | <https://github.com/heng-mu/FootTrack> |
| TF-COMB (v1.1) | Bentsen et al. (Bentsen et al., 2022) | <https://github.com/loosolab/TF-COMB> |
| IGV (v2.16.1) | Thorvaldsdóttir et al. (Thorvaldsdottir et al., 2013) | http://www.broadinstitute.org/igv |
| R (v4.3.3 or higher) | N/A | [https://www.r-project.org](https://www.r-project.org/) |
| Other | | |
| Ni-NTA Agarose Beads | QIAGEN | Cat# 30210 |
| VAHTS DNA Clean Beads | Vazyme | Cat# N411-02 |
| Concanavalin A-coated magnetic beads | Bangs Laboratories | Cat# BP531 |

**Cell culture**

All cells were cultured according to standard procedures.

HepG2 cells (Procell) were maintained in MEM with NEAA (Procell) and K562 cells (Procell) were maintained in RPMI 1640 (Thermo Fisher Scientific).

Mouse embryonic fibroblasts (MEFs) were derived from 13.5 days mouse embryos (ICR), then maintained in DMEM-high glucose (Life Technologies) supplemented with 10% fetal bovine serum (FBS, Gibco), 1 mmol/L L-glutamine (Thermo Fisher Scientific). Puromycin-resistant MEF cells were derived from 13.5 days mouse embryos (DBA and C57 background), and puromycin-resistant feeder were obtained by treating these MEFs with mitomycin C for 3 h.

mESC medium including DMEM-high glucose medium, supplemented with 15% FBS, 1 mmol/L L-glutamine, 100× nucleosides (EMD Millipore), 100× NEAA (Millipore), 0.11 mmol/L 2-mercaptoethanol (Sigma-Aldrich), 10^3^ U/mL LIF (Millipore) and 100× penicillin/streptomycin (Gibco). mESC were cultured on feeder, which was obtained by treating MEF cells (ICR) with mitomycin C for 3 h.

The mESC cell line R1 (includes wild-type and knockout cell lines) were cultured in mESC medium supplemented with 1 μmol/L PD0325901 (Selleck) and 3 μmol/L CHIR99021 (Selleck), while mESC-OG2 cell line used in BRM014 treatment experiment maintained in mESC medium. The OG2 cell line is previously generated in Gao lab(Wang et al., 2022).

**Cell line generation**

We established rtTA-Cas9-EGFP mESC lines and selected three Yy1 sgRNA sequences, which were cloned into the lentiGuide vector (Addgene). Each sgRNA-containing vector (Table S6) included markers for BFP, RFP, and puromycin resistance, respectively. The lentiGuide-Yy1 sgRNA plasmids were extracted and purified using the EndoFree Plasmid Maxi Kit (CoWin Biotech Co.).

To produce lentivirus, the vectors were transfected into 293T cells along with packaging plasmids psPAX2 and pMD2G. Harvested lentivirus after transfection 72 h, then infected 3×10^4^ rtTA-Cas9-EGFP mESCs with three types lentiGuide viruses simultaneously. After 8-10 h of virus infection, cells were washed with culture medium to remove residual virus and then maintained in fresh medium.

Following lentiviral infection, cells were selected with puromycin for 7 days, during the selection, mESC were cultured on the puromycin resistant feeder. BFP and RFP-positive mESC were sorted using the CytoFLEX SRT Cell Sorter (Beckman Coulter). Subsequently, Cas9 protein expression induced by doxycycline and Yy1 knockout efficiency was confirmed by Western blot.

**Purification of SsdA_tox_, Ddd_Ss, and Ddd_Fa for deamination assay**

The expression and purification of SsdA_tox_ were performed as previously described, with minor modifications (de Moraes et al., 2021). The pETduet-SsdA_tox_-SsdAI vector was introduced into Rosetta™ 2 (DE3) Singles™ Competent Cells (EMD Millipore). After overnight culture, transformed cells were inoculated into 4 liters of 2YT medium (1:100 dilution) and grown to approximately OD600 = 0.6. Protein expression was induced with 0.5 mM IPTG (Sigma-Aldrich), followed by incubation at 16°C for 18 h. Cells were harvested by centrifugation (4,000 rpm, 10 min) and resuspended in lysis buffer (50 mM Tris-HCl pH 7.4, 500 mM NaCl, 10 mM imidazole, 5 mM beta-mercaptoethanol, 100 uM PMSF, 1× protease inhibitor cocktail). Lysis was performed using a high-pressure cell cracker (JN-miniPro), and the lysate was clarified by centrifugation (28,000 g, 30 min). The his-tagged SsdA_tox_-SsdAI complex in the supernatant was purified using Ni-NTA beads (QIAGEN) by incubation at 4°C for 3 h. Then beads were washed by lysis buffer 3 times before the denaturation. To separate SsdA_tox_ from SsdAI, the beads underwent denaturation in denaturation containing 8 M urea for 16 h at 4°C, followed by renaturation through successive washes with lysis buffer containing decreasing urea concentrations (6 M, 4 M, 2 M, 1 M, 0 M). The refolded proteins bound to Ni-NTA beads were eluted by Elution buffer (50mM Tris-HCl pH7.4, 500mM NaCl, 300mM imidazole, 1mM DTT, 5% glycerol). Imidazole was subsequently removed by sequentially dialysis against dialysis buffer containing 500 mM NaCl (50 mM Tris-HCl pH 7.4, 500 mM NaCl, 1 mM DTT, 10% glycerol) and dialysis buffer containing 200 mM NaCl. Purity and quantification was assessed by SDS-PAGE, and high-quality fractions were stored at -80°C.

The expression and purification of Ddd_Fa and Ddd_Ss were performed as previously described(Mi et al., 2023), with minor modifications. The pCOLADuet1-10His-Ddd_Fa-com, pCOLADuet1-10His-Ddd_Ss-com vectors (gift from Yangming Wang’s lab in Peking Univerisity) were introduced into Rosetta™ 2 (DE3) Singles™ Competent Cells (EMD Millipore). The steps for culture, collection, lysis and protein bound with Ni-NTA beads are the same as purification of SsdA_tox_. To separate Ddd_Fa and Ddd_Ss from its immunity protein, the beads underwent denaturation in lysis buffer containing 6M GuHCl for 3 h at 4°C, followed by renaturation through successive washes by lysis buffer containing 10uM ZnCl_2_ with decreasing GuHCl concentrations (6 M, 5 M, 4 M, 3 M, 2 M, 1 M, 0M). The steps of elution, dialysis and quantification is the same as the protocol of SsdA_tox_.

**Deamination assay to analyze the deaminase activity on oligo and naked genomic DNA**

DNA deamination assays on oligonucleotides were performed as previously described (Mok et al., 2020, de Moraes et al., 2021), with some modifications. All DNA substrate (Table S6) were purchased from Sangon Biotech (Shanghai, China) and contained a 6-FAM fluorophore at the 5′ end for visualization. To generate double-stranded (ds) DNA substrate, 6-FAM-labeled oligonucleotides were annealed with their complementary unmodified strands at a final concentration of 10 μM in 1x annealing buffer (10 mM Tris pH 7.5, 50 mM NaCl, 1 mM EDTA). Deamination reactions were carried out in a 10 μL mixture consisting of 10 mM Tris-HCl pH 7.4, 0.1 μM substrate, and deaminase at the concentrations indicated in Fig. S1. The reactions were incubated at 37°C for 1 hour. To terminate the deamination reaction, samples were heated at 85°C for 15 min, followed by the addition of 0.3 μL UDG (NEB) and further incubation at 37°C for 30 min. Substrate cleavage was induced by adding 0.5 μL of 2M NaOH and incubating at 95°C for 10 min. The mixture was then cooled on ice, combined with 10 μL of formamide and 2 μL of 6× purple DNA loading dye (NEB), and analyzed by 15% 8M urea-denaturing acrylamide gel electrophoresis in 0.5× TBE buffer. The 6-FAM signal was detected and quantified using the ChemiDoc MP imaging system (Bio-Rad).

For deaminase activity test on naked genomic DNA, reactions were performed in 20 μl deamination mixture containing 10 mM Tris-HCl pH 7.4, 1× PIC, 1 mM DTT, 0.8 ng lambda DNA, 0.1 U/μl Uracil Glycosylase Inhibitor (UGI, NEB), 80 ng genome DNA extracted from R1 cells, and deaminase at the concentrations indicated in Fig. S1. Reactions were incubated for 10 min at 37°C. DNA was precipitated by adding 20 μL of isopropanol and 2 μL of 3M NaAc, followed by incubation at -80°C for 1 h. The DNA pellet was then resuspended in 20 μL of ddH₂O. 10 ul DNA were used for genome-wide library preparation and sequencing, following the cFOOT-seq protocol.

**UPLC-MS/MS analysis for testing deaminase activity on DNA with C and 5mC**

DNA substrate containing cytosine (C-DNA) or 5-methylcytosine (5mC-DNA) were prepared via PCR amplification using either dCTP or 5-methyl-dCTP (5m-dCTP) to replace dCTP. The PCR was performed with primers C-DNA/5mC-DNA_F and C-DNA/5mC-DNA_R, using C-DNA/5mC-DNA_template as the template and TaKaRa Taq™ Hot Start Version (TaKaRa) for amplification. The PCR product was purified using the SanPrep Column DNA Gel Extraction Kit (Sangon), following the manufacturer's instructions. The sequences of the primers and template are described in Table S6.

For the deamination test, 40 ng of PCR-amplified DNA substrate was incubated with increasing concentrations of deaminase (0, 200, 400, 800, 1600, 3200 nM) in a 10 μL reaction mixture containing 10 mM Tris-HCl, 1× PIC, 1 mM DTT, and 0.1 U/μl UGI. The reaction was incubated at 37°C for 10 min with continuous mixing at 1000 RPM. After deamination, DNA was precipitated by adding 10 μL of isopropanol and 1 μL of 3 M NaAc, followed by incubation at -80°C for 1 h. The DNA pellet was then resuspended in 20 μL of ddH₂O.

To measure the deamination efficiency, the purified deaminated DNA was first digested into single nucleosides by nuclease P1 (NEB) and then dephosphorylated by Quick CIP (NEB) according to the manufacturer’s instructions. The nucleoside composition of the DNA samples was detected by ACQUITY UPLC system (Waters) and Triple Quad™ 6500+ LC-MS/MS system (SCIEX) using multiple reaction monitoring (MRM) mode with an ACQUITY Premier HSS T3 (100 Å, 1.8 μm, 2.1 × 100 mm, Waters) column. The flow rate was set at 0.3 mL/min with mobile phases A (water with 0.1% formic acid) and B (100% methanol). The linear gradient was as follows: 100% to 95% A (0-2 min), 95% to 90% A (2-4 min), 90% to 50% A (4-7 min), 50% to 5% A (7-7.5 min), 5% A (7.5-9 min), 5% to 100% A (9-9.1 min) and 100% A (9.1-10 min). The quantifier transitions for each nucleoside were: dC: 228.1/112.1 (CE 20, DP 20); dG: 268.1/152.1 (CE 20, DP 60); 5mC: 242.1/126.1 (CE 17, DP 20). And retention time for each compound were: 5mC: 3.75 min; dC: 2.38 min; dG: 5.05 min. All compounds were measured in positive ESI mode and were quantified by interpolating the peak areas of the quantifier MRM transitions from the standard curves.

**cFOOT-seq procedures.**

*Nuclei preparation*: Collected 5×10^4^ cells for each sample, washed with cold DPBS-0.04% bovine serum albumin (BSA), centrifuge at 300g, 4°C, 5min, remove supernatant. The pellets were resuspended in 50 μl cold nuclei extraction buffer (modified Omni-ATAC buffer: 10 mM Tris-HCl, 10 mM NaCl, 3 mM MgCl2, 0.1% Tween-20, 0.1% IGEPAL CA-630, 0.01% Digitonin, 0.1 mM EDTA, 1× PIC, 1%(w/v) BSA). Incubate on ice 3min, then add 1ml cold stop buffer (10 mM Tris-HCl, pH 7.4, 10 mM NaCl, 0.1mM EDTA, 1×PIC, 1%(w/v) BSA) and invert tube 3 times gently mix. The nuclei were collected through centrifugation at 700g, 4°C, 10 min. Carefully remove the liquid above without disturbing the sediment.

*Deaminase Reaction*: Resuspend the pellet gently in 50 μl deamination mix (10 mM Tris-HCl, 1× PIC, 1 mM DTT, 2 ng lambda DNA, 0.1 U/μl UGI, 5-15U/μl SsdA_tox_), incubate at 37°C for 10 min with shaking at 1000 RPM. The concentration of SsdA_tox_ required for each cell lines should be tested to generate general genomic conversion rate around 30-35%. Longer incubation increases the conversion rate but may compromise footprint detection due to potential loss of TF occupancy on chromatin.

*DNA Extraction*: After the reaction, add 10% SDS and 1 M NaHCO_3_ to final 250 μl with 1% SDS and 0.1M NaHCO_3_, and incubate at 65°C for 1.5h with shaking at 1350 RPM. Then add 250 μl PCI and mix by full-speed vortexing for 5s. Remove the upper liquid to a new tube and then precipitate DNA with equal volume isopropanol.

*Genome-Wide Library Preparation and Sequencing*: Fragment genomic DNA using Covaris DNA shearing (peak power: 50.0, duty factor: 20.0, cycles/burst: 200, duration: 25 sec). Prepare the DNA library using the EpiArt DNA Methylation Library Kit for Illumina V3 (Vazyme) as described. Briefly, 20 ng of input genomic DNA was denatured to single-stranded DNA, followed by the ligation of a truncated linker at the 3' end. The single-stranded DNA was then extended to double-stranded DNA using extension primers. After purification with 1.2× VAHTS DNA Clean Beads (Vazyme, N411), a truncated adapter was ligated to the 5' end of the original template strand of the double-stranded DNA. The reaction products were further purified with 1× VAHTS DNA Clean Beads, and the complete library was amplified with universal i5 and i7 primers for 7 cycles. Then the complete library was purified by 0.85× VAHTS DNA Clean Beads, and eluted by 10-20ul elution buffer.

Finally, paired-end 150 bp sequencing was performed on the Illumina NovaSeq 6000 system. For condition test, approximately 7 million read pairs per sample (~1.5× depth) are sufficient. For de novo footprint prediction, approximately 100 million read pairs per sample (~6.5× depth) are recommended for downstream analyses. More details can be found in Table S8.

**cFOOT-seq procedures for small number of cells with ConA beads**

*Nuclei preparation:* Collect the appropriate number of cells, wash with 500 μl wash buffer (20 mM HEPES, 150 mM NaCl, 0.5 mM Spermidine, 0.1% BSA, 1 × PIC). For cell counts between 1000 and 5000, dilute as needed, and for 5 to 200 cells, a stereomicroscope may be used. Resuspend the cells with 300 μl binding buffer (20 mM HEPES-KOH, 10 mM KCl, 1 mM CaCl_2_, 1 mM MnCl_2_). After resuspending 5 μl of Concanavalin A-coated magnetic beads with 15 μl binding buffer, mix with the cell suspension and incubate for 10 min, place the EP tube on a magnetic stand for 1 min, discard the liquid and remove the EP tube, then use 500 μl of cold blocking buffer (20 mM HEPES, 150 mM NaCl, 0.5 mM Spermidine, 0.1% BSA, 2 mM EDTA, 1× PIC, 0.01% digitonin) to resuspend the beads and incubate for 5 min. Repeat the magnetic separation and liquid removal. Subsequently, resuspend the beads in 500 μl of cold dig-Wash Buffer (20 mM HEPES, 150 mM NaCl, 0.5 mM Spermidine, 0.1% BSA, 1× PIC, 0.01% digitonin) and wash twice more using the same procedure.

*Deaminase Reaction and DNA Extraction*: Resuspend the beads with 100 μl reaction buffer (10 mM Tris-HCl pH=7.4, 1× PIC, 1 mM DTT, 2 ng lambda DNA, 0.1 U/μl UGI, 2-10 U/μl SsdA_tox_) ,incubate at 37 °C, shaking at 1000 rpm for 10 min. Add 12.5 μl of NaHCO_3_ and 12.5 μl of 10% SDS, mix and incubate at 65 °C, shaking at 1350 rpm for 1 h. Place the EP tube on the magnetic stand for 1min, and transfer the liquid to a new EP tube . Add 125 μl of PCI and vortex at full speed for 5 seconds to mix. Carefully transfer the upper liquid to a new tube and precipitate the DNA with an equal volume of isopropanol.

*Genome-Wide Library Preparation and Sequencing*: The procedure closely follows that of cFOOT-seq, with slight modifications. Given the low quantity of genomic DNA, the entire sample is used for fragmentation. The fragmented DNA is then enriched using VAHTS DNA Clean Beads beads before denaturation and subsequent ligation of the 3' adapter with the EpiArt DNA Methylation Library Kit for Illumina V3 (Vazyme).

**cFOOT and ATAC-seq combination procedures.**

There are two protocols included ATAC-cFOOT-seq and cFOOT-ATAC-seq. Two shared steps of Tn5 transposase generation, and DNA library preparation steps are done as below.

*Tn5 Transposome Generation with Cytosine-Free Adaptor*: To avoid cytosine deamination of Tn5 adapters by SsdA_tox_, a cytosine-free Tn5 adapter was prepared by annealing Tn5 Primer D (Table S6), which lacks cytosine in its 5' overhang, with the universal Tn5 ME oligo (5‘-phos-CTGTCTCTTATACACATCT-NH2-3’). The annealing was performed in a thermal cycler with an initial denaturation at 95°C for 5 minutes, followed by a gradual cooling to 10°C at a rate of -0.1°C/s. Subsequently, 5 µM of the cytosine-free adaptors were incubated with unloaded Tn5 transposase protein in coupling buffer (Vazyme, S601) at 30°C for 1 hour, producing a final concentration of 750 nM Tn5 transposome. The loaded Tn5 transposase can be stored at -20°C for up to 1 year.

*DNA library preparation for combined procedures:* DNA library was constructed with modification of protocols used in the EpiArt DNA Methylation Library Kit for Illumina V3 (Vazyme). In brief, denature 50 ng genomic DNA, ligate a truncated linker at the 3' end, extend to full duplex, and purify with 1.2 x VAHTS DNA Clean Beads. Then purified DNA is initially amplified for 4 cycles with universal i7 primers and i5_bridge primer (Table S6) to add 5’ sequencing fragment. After purification by 1× VAHTS DNA Clean Beads, the library is further amplified with universal i5 and i7 primers for 8 cycles, which can match illumine next generation sequencing platform. Finally, the library fragments in the size range of 300-500 bp can be recovered by agarose gel electrophoresis to obtain the final library.

**ATAC-cFOOT-seq protocol:**

*Nuclei preparation*: The operation is same as that in cFOOT-seq.

*Tn5 transposase reaction*: Resuspended nuclei pellet gently with 50 μl tagmentation mix (10mM Tris-HCl pH=7.6, 5mM MgCl_2_, 10% Dimethylformamide (DMF), 33%(v/v) DPBS, 100nM Tn5 transposome with cytosine-free adaptor), incubate reaction at 37°C for 15min in a mixer with 1000RPM.

*Deaminase Reaction*: Stop Tn5 reaction by adding 500 μl cold tagmentation stop buffer (10mM Tris-HCl pH=7.4, 1mM DTT, 1x PIC), mix well, collect nuclei through centrifugation at 700g, 4°C, 5min. Remove all supernatant, resuspension pellet by deamination mix (10mM Tris-HCl pH=7.4, 1× PIC, 1mM DTT, 0.2ng lambda DNA with Tn5 adaptor sequence, 0.1 U/μl UGI, 7.5-15U/μl SsdA_tox_), incubate reaction at 37°C for 10 min in a mixer with 1000 RPM.

*DNA Extraction and library preparation*: The operation of DNA extraction is same as that in cFOOT-seq. DNA library is prepared as described in DNA library preparation for combined procedures.

*Sequencing*: paired-end 150 bp sequencing was performed on the Illumina NovaSeq 6000 system. For condition testing, approximately 3 million read pairs per sample (~6× depth in open chromatin regions) are sufficient. For de novo footprint prediction, approximately 60 million read pairs per sample (~42× depth in open chromatin regions) are recommended.

**cFOOT-ATAC-seq protocol:**

*Nuclei preparation*: The operation is same as that in cFOOT-seq.

*Deaminase reaction*: Resuspended nuclei pellet by 50 μl deamination mix (10mM Tris-HCl pH=7.4, 1× PIC, 1mM DTT, 33%(v/v) DPBS, 0.02% BSA, 0.1 U/μl UGI, 1-5U/μl SsdA_tox_), incubate reaction at 37°C for 10 min in a mixer with 1000 RPM.

*Tn5 transposase reaction*: Stop deaminase reaction by adding 500 μl cold deamination stop buffer (10mM Tris-HCl pH=7.4, 1× PIC, 1mM DTT, 33%(v/v) DPBS, 0.02% BSA, 5mM MgCl_2_). Collect nuclei through centrifugation at 700g, 4°C, 10 min. Remove supernatant, resuspension pellet by 50 μl tagmentation mix (10mM Tris-HCl pH=7.6, 5mM MgCl_2_, 10% Dimethylformamide (DMF), 33%(v/v) DPBS, 100nM Tn5 transposome with cytosine-free adaptor). Incubate reaction at 37°C for 15-30 min in a mixer with 1000 RPM.

*DNA Extraction and library preparation*: The operation of DNA extraction is same as that in cFOOT-seq. DNA library is prepared as described in DNA library preparation for combined procedures.

*Sequencing*: paired-end 150 bp sequencing was performed on the Illumina NovaSeq 6000 system. For condition testing, approximately 3 million read pairs per sample (~2× depth in open chromatin regions) are sufficient. For de novo footprint prediction, approximately 60 million read pairs per sample (~13× depth in open chromatin regions) are recommended.

**Single cell ATAC-cFOOT-seq procedures**

*Nuclei preparation and deaminase reaction*. Follow the same steps as in ATAC-cFOOT-seq.

*DAPI staining and FACS sorting*: After the deaminase reaction, collect nuclei through centrifugation at 700 g, 4°C, 5 min. Remove all supernatant, resuspend the pellet in DPBS-0.5% BSA containing DAPI (1:1000) and incubate in the dark on ice for 15 min. After incubation, centrifuge and remove all supernatant, resuspend the nuclei in DPBS-0.5% BSA without DAPI to prepare for sorting. Then sort the DAPI-positive single nuclei into single wells of a 96-well plate containing 1μl lysis buffer (10 mM Tris pH 8.0, 20 mM NaCl, 1 mM EDTA, 0.1% SDS, 500 nM Carrier ssDNA, 60 μg/mL protease K (QIAGEN)) by CytoFLEX SRT Cell Sorter (Beckman Coulter)**.** After sorting, immediately seal the single-cell lysis plate and centrifuge at 1,000 g for 1 min at RT to avoid losing the cells on the plate wall.

*Nuclei lysis and Tn5 release*: After centrifugation, incubate the single-cell lysis plate at 65 °C for 15 min, 95°C 2 min in a thermal cycler with a heated lid set to 80 °C. Then add 1 μl 3% Triton X-100 to quench SDS and stop the lysis reaction. After this, store the plate at -80 °C or proceed to downstream amplification.

*Library preparation*: The DNA library was constructed following a modified protocol based on the EpiArt DNA Methylation Library Kit for Illumina V3 (Vazyme). Briefly, single-cell genomic DNA was denatured in the plate, and a truncated linker was ligated to the 3' end. The ligated DNA was amplified for 8 cycles using universal i7 primers and i5_bridge primers with well-specific barcodes to distinguish single cells in each well. The amplified products from all wells were pooled and purified twice using 1× magnetic beads. The library was then further amplified for 12 cycles with universal i5 and i7 primers to ensure compatibility with the Illumina next-generation sequencing platform and purified using 0.85× magnetic beads. To achieve a total DNA yield of 300-500 ng, an additional 6 cycles of amplification are required, followed by purification using 0.85× beads. Finally, library fragments in the size range of 300-500 bp were recovered by agarose gel electrophoresis to obtain the final library.

*Sequencing*: paired-end 150 bp sequencing was performed on the Illumina NovaSeq 6000 system, with approximately 2 million read pairs per cell. More details can be found in Table S2.

**OSKM reprogramming**

The reprogrammable MEF cell line was generated in previous study (Xi et al., 2022), doxycycline was added to induce MEF cells overexpress OSKM at a final concentration of 1 µg/ml. Samples were collected at 24, 48, and 96 h after doxycycline induction.

**BRM014 treatment**

BRM014 compounds (BRM/BRG1 ATP Inhibitor-1, MedChem Express) dissolved in dimethyl sulfoxide (DMSO) to final concentration of 10 mM. Add the required concentration directly to the well and shaken thoroughly.

For the recovery, the medium was used to gently flush the well 3 times and finally to medium without BRM014.

**cFOOT-seq reads preprocessing**

The raw sequencing reads obtained from next-generation sequencing were first evaluated for quality using FastQC v0.12.1. Subsequent trimming of these reads was performed with Trim Galore v0.6.10, employing specific parameters to enhance both quality and utility for downstream analysis. These parameters included --trim-n, --clip_R1 3, --clip_R2 10, --three_prime_clip_R1 3, --three_prime_clip_R2 3, --length 35, -q 20, --fastqc, --paired. The alignment of the trimmed reads was conducted using BASAL (<https://github.com/JiejunShi/BASAL>) (Xu et al., 2025), which is specifically designed for mapping nucleotide base-conversion sequencing reads in a conversion-sensitive manner. The alignment parameters were set to -m 1, -x 1000, -r 1, -v 0.06, -s 16, -S 1, -n 0, -g 1, -M C:T. For further processing, the BasalKit (Xu et al., 2025) ‘avgmod’ module was utilized with parameters -r -m 1 -i correct to process the aligned reads, producing a TSV file that recorded the average C-to-T conversion rate for each cytosine. In refining the analysis, SNP sites were systematically excluded. For detailed analysis of TF occupancy, the study proceeded with two analytical approaches: analysis based on known transcription factor binding sites and de novo prediction of transcription factor binding sites.

**FootTrack analysis.**

To comprehensively assess transcription factor occupancy and dynamics across variable conditions, we developed the FootTrack (Footprint Analysis for Tracking TF Occupancy and Kinetics, https://github.com/ZhangLab-TJ/FootTrack). Constructed on the basis of TOBIAS (Bentsen et al., 2020), FootTrack serves as an extensive analytical platform tailored for both the analysis of known transcription factor binding sites and the prediction of new sites. The tool’s structured analytical process comprises three main components: bias correction, analysis of known transcription factor binding sites, and de novo prediction of transcription factor binding sites. Together, these elements enable a robust examination of transcription factor interactions within the genomic landscape.

1. **Bias correction**

To assess enzymatic bias, data from naked DNA samples with a conversion rate of approximately 40% were utilized, as this rate closely aligns with the conversion rate observed in the open chromatin regions of cFOOT-seq. Bias was quantified by aggregating conversion rate across different sequence contexts and normalizing them by the frequency of each context in the background. A position-weight matrix (PWM) was generated using the sequence context within a ±10 bp window, and this PWM was subsequently applied in further analyses to compute bias for each context.

For the correction process, FootTrack first calculates the background conversion rate based on the observed data. This background rate is then multiplied by the context bias to obtain the expected conversion rate for each base. The corrected conversion rate is determined by subtracting the expected conversion rate from the observed value (Bentsen et al., 2020).

Two background calculation modes are available. The first, the global background, uses the average conversion rate across the entire genome, preserving both chromatin accessibility and transcription factor binding information. The second, the local background, calculates the average conversion rate within a ±50 bp window around each base (Koohy et al., 2013). This mode improves TF footprint detection by reducing biases from regional chromatin accessibility variations. The correction formula is as follows:

Global background: $\hat{x}_{i}=\frac{1}{n}\sum_{1}^{n} x_{j}$; Local background: $\hat{x}_{i}=\frac{1}{101}\sum_{j=i-50}^{i+50} x_{j}$;

$$e_{i}=\hat{x}_{i}*b_{i}$$

$$c_{i}=x_{i}-e_{i}$$

Where:

$x_{i}$ is the observed conversion rate

$i$ is the respective position in the genome

n is the total number of C/G in genome

$e_{i}$ is the expected conversion rate

$b_{i}$ is the calculated bias level

$c_{i}$ is the corrected conversion rate

The corrected conversion rate are then used in subsequent analyses to improve the accuracy of transcription factor binding site identification and characterization.

1. **Analysis based on known transcription factor binding sites.**

Potential TF binding sites are identified by scanning motifs in the peak regions defined by ChIP-seq, with consideration of strand orientation. The flanking regions are defined as extending 50 bp on either side of each motif. The FOS at each site is calculated by subtracting the conversion rate in the flanking regions from that at the motif site. Averaging the FOS across all binding sites or specific regions gives the Transcription Factor Occupancy Score (TFOS), which reflects overall TF occupancy. The data sources for TF binding information are provided in Table S1.

1. **Analysis based on de novo prediction of transcription factor binding sites.**

FootTrack provide two strategies for footprint detection and motif prediction:

1) For cFOOT-seq data, we propose strategy 1 (S1), adopted from TOBIAS(Bentsen et al., 2020). S1 calculates footprint scores for each motif with the corrected data, and applies statistical methods to identify motifs with higher scores as potential TF binding sites in interested regions.

*Footprint Scoring calculation*: FootTrack employs footprint scoring to predict TF binding sites by assessing the likelihood of TF binding on genome. The footprint score is computed by analyzing the mean conversion rate of two strategically defined genomic regions: the 'center' and the 'flanks.' Specifically, the 'center' is defined by the interval $[i + w_{f} , i +w_{f} + w_{c}]$, with $w_{f}$, the flank width, set by default to 20 nucleotides, and $w_{c}$, the center width, to 10 nucleotides. The 'flanks' are the regions extending from $i$ to $i + w_{f}$ and from $i +w_{f} + w_{c}$ to $i +{2 w}_{f} + w_{c}$. The footprint score is determined by subtracting the mean conversion rate of the 'center' from that of the 'flanks'.

*TF Binding Site Detection*: FootTrack uses the MOODS (Korhonen et al., 2017) ([https://github.com/jhkorhonen /MOODS](https://github.com/jhkorhonen%20/MOODS)) to scan for motifs, applying a p-value threshold of 1e-4. For each identified TF motif, footprint scores are calculated and matched accordingly. Background base pair probabilities are estimated based on the open chromatin regions, and a background distribution of scores is generated by randomly subsetting these regions at ~200 bp intervals. Each TF motif is then classified into bound and unbound sites based on a score threshold. This threshold is determined by the significance level derived from a normal distribution fit to the background score distribution, with a p-value cutoff of 0.05. Open regions are usually used for de novo prediction, considering the enrichment of TF binding.

2) For ATAC-cFOOT-seq and cFOOT-ATAC-seq, we proposed strategy 2 (S2), conceptionally adopted from Footprint tools (Vierstra et al., 2020). S2 initially detects footprint regions by binomial statistical test and then scans for motifs within the identified footprint regions to predict TF binding sites.

*Footprint Detection*: The BasalKit (Xu et al., 2025) ‘avgmod’ module is used to extract data from the mapped BAM files, providing the number of reads that cover each base and the number of converted reads at each base. The expected conversion rate for each base is derived from the previous bias correction step. The number of converted reads at each base is treated as the number of successes in a series of independent trials, allowing the observed data to be modeled using a binomial distribution. By comparing the observed data to the expected data, we assess whether the observed conversion rate is significantly lower than expected. Specifically, the null hypothesis (H_0_) posits that the observed conversion rate at each base equals the expected. The alternative hypothesis (H_1_) suggests that the observed conversion rate is significantly lower than expected, indicating potential protein binding at the corresponding position. In our analysis, a threshold of p<0.05 is used to classify a position as likely to be a footprint. Since deamination occurs only at C/G sites, and to minimize the proportion of missing values, we extend the footprint by 5 bp on either side to define the final footprint region.

*TF Assignment*: Similar to the approach used in S1, once TF motifs within open chromatin regions are identified, any motif that overlaps with a footprint by more than 50% is considered a potential transcription factor binding site

1. **Differential transcription factor binding analysis**

FootTrack employs volcano plots to visualize differences in TF occupancy between different cells and conditions. TFs are selected based on significant differential footprint scores greater than 0.1 and a -log10 (p-value) exceeding the 90th percentile, identifying them as condition-specific TFs. To minimize false positives due to motif similarity, the TOBIAS ClusterMotifs tool is used to group motifs by similarity, with a threshold of 0.4 for robust clustering. Each cluster is then systematically renamed for clarity, reducing redundancy from overlapping motif characteristics (Table S7). In the volcano plot, each cluster is represented by the point with the highest differential binding score, highlighting the most significant TF binding changes.

**Performance evaluation**

The performance of strategies S1 and S2 across different techniques was evaluated by calculating the Area Under the ROC Curve (AUC) using both local and global background modes. During AUC calculation, the footprint score was used as the observed value in S1, while the number of overlapping bases between the footprint and the motif was used as the observed value in S2. The ground truth for bound TF binding sites was defined as motifs reside in the ±50 bp window centered by the corresponding ChIP-seq peak summits. AUC values were calculated for 50 TFs with strong binding signals and compared to assess the predictive performance of each strategy across cFOOT-seq, cFOOT-ATAC-seq, and ATAC-cFOOT-seq.

**Single-Cell Data Analysis**

For the single-cell analysis, raw FASTQ files were processed using UMI-tools to tag reads based on the barcode. Following this, the reads were mapped to the reference genome using standard bulk analysis methods. After mapping, the reads were split according to their barcode. The BasalKit (Xu et al., 2025) ‘avgmod’ module was used for downstream analysis of each cell, providing information on the conversion rate and depth for each cell. The merged dataset was then used as the reference for downstream analyses, including calculating the Fraction of Reads in Peaks (FRiP) and other statistical metrics.

For each cell, the data were subsequently corrected using the FootTrack. To visualize the TF binding, the corrected conversion rate at motifs identified within ChIP-seq peaks for each TF were plotted. To visualize the TF binding patterns at individual loci, a 5-bp smoothing window was applied to the data. Cells with more than 80% missing values were excluded from further analysis.

**Single-Molecule Data Analysis**

Single-molecular footprint analysis provides a more sensitive quantification of TF occupancy at specific loci. For each footprint of interest, reads fully spanning the region were extracted, and the conversion rate of C and G within the footprint were quantified. For the positive strand, footprints were considered unoccupied if the proportion of cytosines converted in the footprint exceeded 10%. Similarly, for the negative strand, footprints were classified as unbound if the proportion of guanines converted exceeded 10%. This approach enables precise assessment of TF binding dynamics at single-molecule resolution.

**ChromHMM analysis**

We use the ChromHMM file of HepG2 from ENCODE (accession number: ENCFF808IZE), which contains 18 chromatin states, such as TssA, Tssflank, Tx, TXWk and other regions. For better clustering, we merge TssFlnk, TssFlnkU and TssFlnkD as Tss flank region, EnhG1 and EnhG2 as EnhG region, EnhA1 and EnhA2 as EnhA region, Tx and TxWk as Tx region, for the reason that these regions contain similar histones. Thus, we get 13 chromatin states, and then use K-means clustering method to get the TFOS of transcription factors in different chromatin states.

**Analysis of transcription factor co-occurrence**

To enhance the understanding of transcription factor interactions, TF-COMB (Bentsen et al., 2022) was utilized to analyze the co-occurrence among transcription factors. To minimize the inclusion of false positives in the TF pair analysis, the ChIP-seq motifs with a positive Footprint Occupancy Score (FOS) are used as genuine binding sites for generating the robust TF interacting pairs.

To expand the analysis of TF pairs across various conditions, we integrated cFOOT-seq data with FootTrack for de novo prediction of transcription factor binding sites. To emphasize condition-specific TF pairs and reduce the potential for false positives due to motif similarity, we conducted a comparative analysis of TF pairs across different experimental conditions. This method enhances the specificity of the findings by facilitating the identification of TF pairs that are uniquely significant under specific conditions.

**Consensus clustering of TF with varying sensitivity to BRM014 in HepG2.**

To identify transcription factors with varying sensitivity to BRM014 treatment, we performed consensus clustering on TFs that met stringent filtering criteria. The filtering process involved three steps: (1) TFOS exhibited negative values under any condition across the two replicates are excluded; (2) TFs occurred at least 500 loci overlapping with ATAC ∩ ChIP ∩ SMARC are retained; (3) Outlier filtration, TFOS matrix transform into a matrix of relative changes from Control [Ri = (Ti - T0)/T_0_]. The DB algorithm (Distance-based outlier detection, *DDoutlier* version 0.1.0) was applied to detect and exclude outliers. Following the above three meticulous steps, we identified 134 qualified TFs for consensus clustering (*ConsensusClusterPlus* version 1.64.0) analysis (Wilkerson and Hayes, 2010) (cluster by K-Means, the distance by Euclidean). Notably, the Delta Area revealed an elbow point at k=4, indicating the presence of four distinct clusters with varying response characteristics.

To categorize TFs into BRM014-sensitive and resistant groups independent of their genomic locations. Among the 134 TFs, we segregated the flank change matrix by enhancers and promoters. By repeating the TFOS-based filtering approach, we narrowed down the list to 100 TFs. We combined the enhancer and promoter flank change matrices and performed consensus clustering (K-Means clustering with Euclidean distance).

The resulting four clusters (C1 to C4) exhibited increasing degrees of reduction in chromatin accessibility upon BRM014 treatment. TFs where both enhancer (E) and promoter (P) were classified as C1 were designated as highly resistant TFs. TFs with E and P in C1 or C2 were designated as moderately resistant TFs. TFs displaying E and P in C4 were designated as highly sensitive TFs. Those with E and P intra C3 or C4 were designated as moderately sensitive TFs. All other TFs were categorized as ambiguous.

This nuanced classification scheme provides valuable insights into the differential responsiveness of TFs to BRM014 treatment, highlighting their potential roles in mediating the complex regulatory networks affected by BRM014.

**Assessing TF Regulatory Preferences**

To evaluate the regulatory preferences of different TF classes, we first selected all binding sites with a FOS > 0. Next, we calculated the fraction of promoters (±1 kb) from both HepG2-specific and housekeeping genes that overlapped these sites, and then computed the ratio of these fractions. HepG2-specific genes were obtained from Harmonizome (https://maayanlab.cloud/Harmonizome/), and human housekeeping genes from the Housekeeping and Reference Transcript Atlas (<https://housekeeping.unicamp.br>).

**Motif Association of Footprints**

To assess how many footprints could be linked to known TFs, we first merged adjacent 2-bp windows with footprint scores > 0.05 into contiguous regions of 5–100 bp, designating these as bona fide footprints. Footprints with < 50 % overlap with known motifs in open-chromatin regions were classified as footprints without known motif, while those with ≥ 50 % overlap were classified as footprints with known motif. We then compared the length distributions and footprint scores of these two footprint types to uncover differences in their binding characteristics.

**References:**

BENTSEN, M., GOYMANN, P., SCHULTHEIS, H., KLEE, K., PETROVA, A., WIEGANDT, R., FUST, A., PREUSSNER, J., KUENNE, C., BRAUN, T., KIM, J. & LOOSO, M. 2020. ATAC-seq footprinting unravels kinetics of transcription factor binding during zygotic genome activation. *Nat Commun,* 11**,** 4267.

BENTSEN, M., HEGER, V., SCHULTHEIS, H., KUENNE, C. & LOOSO, M. 2022. TF-COMB - Discovering grammar of transcription factor binding sites. *Comput Struct Biotechnol J,* 20**,** 4040-4051.

DE MORAES, M. H., HSU, F., HUANG, D., BOSCH, D. E., ZENG, J., RADEY, M. C., SIMON, N., LEDVINA, H. E., FRICK, J. P., WIGGINS, P. A., PETERSON, S. B. & MOUGOUS, J. D. 2021. An interbacterial DNA deaminase toxin directly mutagenizes surviving target populations. *Elife,* 10.

KOOHY, H., DOWN, T. A. & HUBBARD, T. J. 2013. Chromatin accessibility data sets show bias due to sequence specificity of the DNase I enzyme. *PLoS One,* 8**,** e69853.

KORHONEN, J. H., PALIN, K., TAIPALE, J. & UKKONEN, E. 2017. Fast motif matching revisited: high-order PWMs, SNPs and indels. *Bioinformatics,* 33**,** 514-521.

MI, L., SHI, M., LI, Y. X., XIE, G., RAO, X., WU, D., CHENG, A., NIU, M., XU, F., YU, Y., GAO, N., WEI, W., WANG, X. & WANG, Y. 2023. DddA homolog search and engineering expand sequence compatibility of mitochondrial base editing. *Nat Commun,* 14**,** 874.

MOK, B. Y., DE MORAES, M. H., ZENG, J., BOSCH, D. E., KOTRYS, A. V., RAGURAM, A., HSU, F., RADEY, M. C., PETERSON, S. B., MOOTHA, V. K., MOUGOUS, J. D. & LIU, D. R. 2020. A bacterial cytidine deaminase toxin enables CRISPR-free mitochondrial base editing. *Nature,* 583**,** 631-637.

RAMIREZ, F., RYAN, D. P., GRUNING, B., BHARDWAJ, V., KILPERT, F., RICHTER, A. S., HEYNE, S., DUNDAR, F. & MANKE, T. 2016. deepTools2: a next generation web server for deep-sequencing data analysis. *Nucleic Acids Res,* 44**,** W160-5.

THORVALDSDOTTIR, H., ROBINSON, J. T. & MESIROV, J. P. 2013. Integrative Genomics Viewer (IGV): high-performance genomics data visualization and exploration. *Brief Bioinform,* 14**,** 178-92.

VIERSTRA, J., LAZAR, J., SANDSTROM, R., HALOW, J., LEE, K., BATES, D., DIEGEL, M., DUNN, D., NERI, F., HAUGEN, E., RYNES, E., REYNOLDS, A., NELSON, J., JOHNSON, A., FRERKER, M., BUCKLEY, M., KAUL, R., MEULEMAN, W. & STAMATOYANNOPOULOS, J. A. 2020. Global reference mapping of human transcription factor footprints. *Nature,* 583**,** 729-736.

WANG, M., ZHAO, K., LIU, M., WANG, M., QIAO, Z., YI, S., JIANG, Y., KOU, X., ZHAO, Y., YIN, J., LI, T., WANG, H., JIANG, C., GAO, S. & CHEN, J. 2022. BMP4 preserves the developmental potential of mESCs through Ube2s- and Chmp4b-mediated chromosomal stability safeguarding. *Protein Cell,* 13**,** 580-601.

WILKERSON, M. D. & HAYES, D. N. 2010. ConsensusClusterPlus: a class discovery tool with confidence assessments and item tracking. *Bioinformatics,* 26**,** 1572-3.

XI, C., SUN, J., XU, X., WU, Y., KOU, X., ZHAO, Y., SHEN, J., DONG, Y., CHEN, K., SU, Z., LIU, D., YE, W., LIU, Y., ZHANG, R., XU, Y., WANG, H., HAO, L., WU, L. & GAO, S. 2022. Mettl14-driven senescence-associated secretory phenotype facilitates somatic cell reprogramming. *Stem Cell Reports,* 17**,** 1799-1809.

XU, M., LIU, X., WANG, M., LUO, T., GAO, Y., LIU, J. & SHI, J. 2025. BASAL: a universal mapping algorithm for nucleotide base-conversion sequencing. *Nucleic Acids Res,* 53.

**Supplemental information**

Document S1: Figures S1-S9

Table S1-S8

Table S1. reference_data_source

Accession information for various TF ChIP-seq, ATAC-seq, and other datasets across different cell lines (HepG2, K562, R1, MEF).

Table S2. scATAC-cFOOT-seq

The barcodes used for single cell library preparation and different metrics of single cell data in scATAC-cFOOT-seq

Table S3. chomHMM_cluster

Group information of 204 TFs in HepG2 cells clustered by their TFOS in regions defined by chromHMM annotations

Table S4. BRM014_TFOS and Flank_change ratio_all sites

Clustering information of 134 TFs based on their dynamic responses to BRM014 treatment, with change ratio of TFOS and Flanking chromatin accessibility at all binding sites of each TFs in HepG2

Table S5. BRM014_Flank change ratio_ Promoter_Enhancer

Clustering information of 100 TFs in HepG2 based on their change ratio of flanking chromatin accessibility on promoter or enhancer.

Table S6. Oligo sequence data

The sequence of oligos used in this study

Table S7. human_mouse_cluster

Clustering of TFs in human and mouse based on the similarity of binding motifs.

Table S8. cFOOT_data

The cFOOT-seq sequencing data generated in this study are listed.

**Supplementary Figures**

**
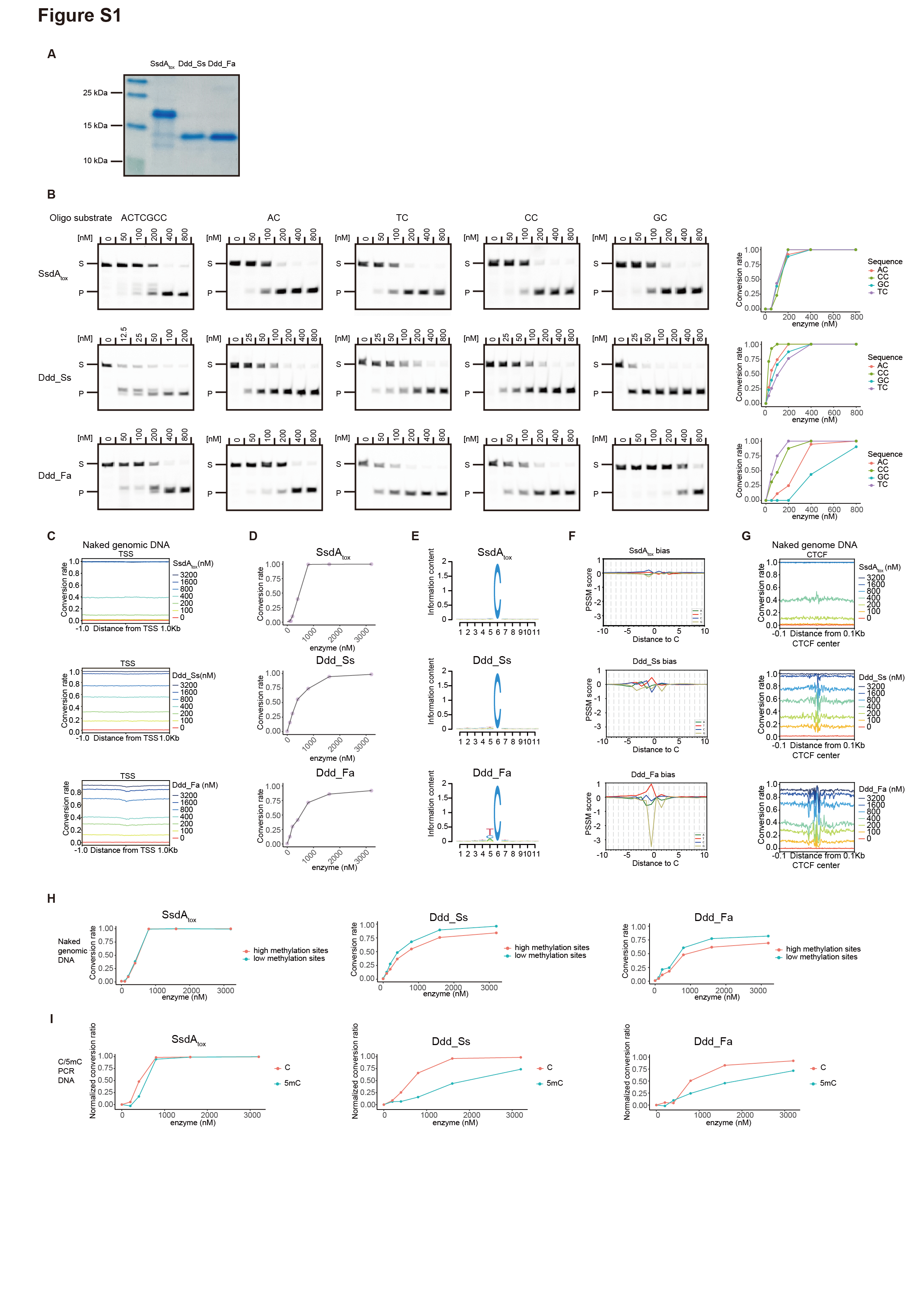
**

**Figure S1: Deaminase activity and sequence bias of SsdA_tox_, Ddd_Ss, and Ddd_Fa across different substrates, related to Figure 1.**

**A.** Gel image showing purified SsdA_tox_, Ddd_Ss, and Ddd_Fa proteins.

**B.** In vitro cytidine deamination assays using various dsDNA oligonucleotide substrates (ACTCGCC, AC, TC, CC, and GC). Left: Gel images showing the products of deamination. Right: Quantification of deamination efficiency for each substrate, treated with SsdA_tox_ (top), Ddd_Ss (middle), and Ddd_Fa (bottom).

**C.** Average conversion rates around transcription start sites (TSS) in R1 naked genomic DNA treated with increasing concentrations of SsdA_tox_ (top), Ddd_Ss (middle), and Ddd_Fa (bottom).

**D.** Average conversion rates in R1 naked genomic DNA treated with increasing concentrations of SsdA_tox_ (top), Ddd_Ss (middle), and Ddd_Fa (bottom).

**E.** Sequence logos displaying the sequences flanking converted cytosines in R1 naked genomic DNA treated with SsdA_tox_ (top), Ddd_Ss (middle), and Ddd_Fa (bottom).

**F.** Sequence bias for SsdA_tox_ (top), Ddd_Ss (middle), and Ddd_Fa (bottom) on R1 naked DNA before bias correction. The position-specific scoring matrix (PSSM) shows nucleotide preferences from -10 to +10 bp relative to cytosine.

**G.** Average conversion rates around CTCF motifs derived from CTCF ChIP-seq peaks in R1 naked genomic DNA treated with increasing concentrations of SsdA_tox_ (top), Ddd_Ss (middle), and Ddd_Fa (bottom).

**H.** Average conversion rates at high methylation sites (top 50% methylation) versus low methylation sites (bottom 50% methylation) in R1 naked genomic DNA treated with increasing concentrations of SsdA_tox_ (left), Ddd_Ss (middle), and Ddd_Fa (right).

**I.** Conversion rates of C and 5mC PCR DNA substrates treated with increasing concentrations of SsdA_tox_ (left), Ddd_Ss (middle), and Ddd_Fa (right), measured by UPLC-MS/MS

**
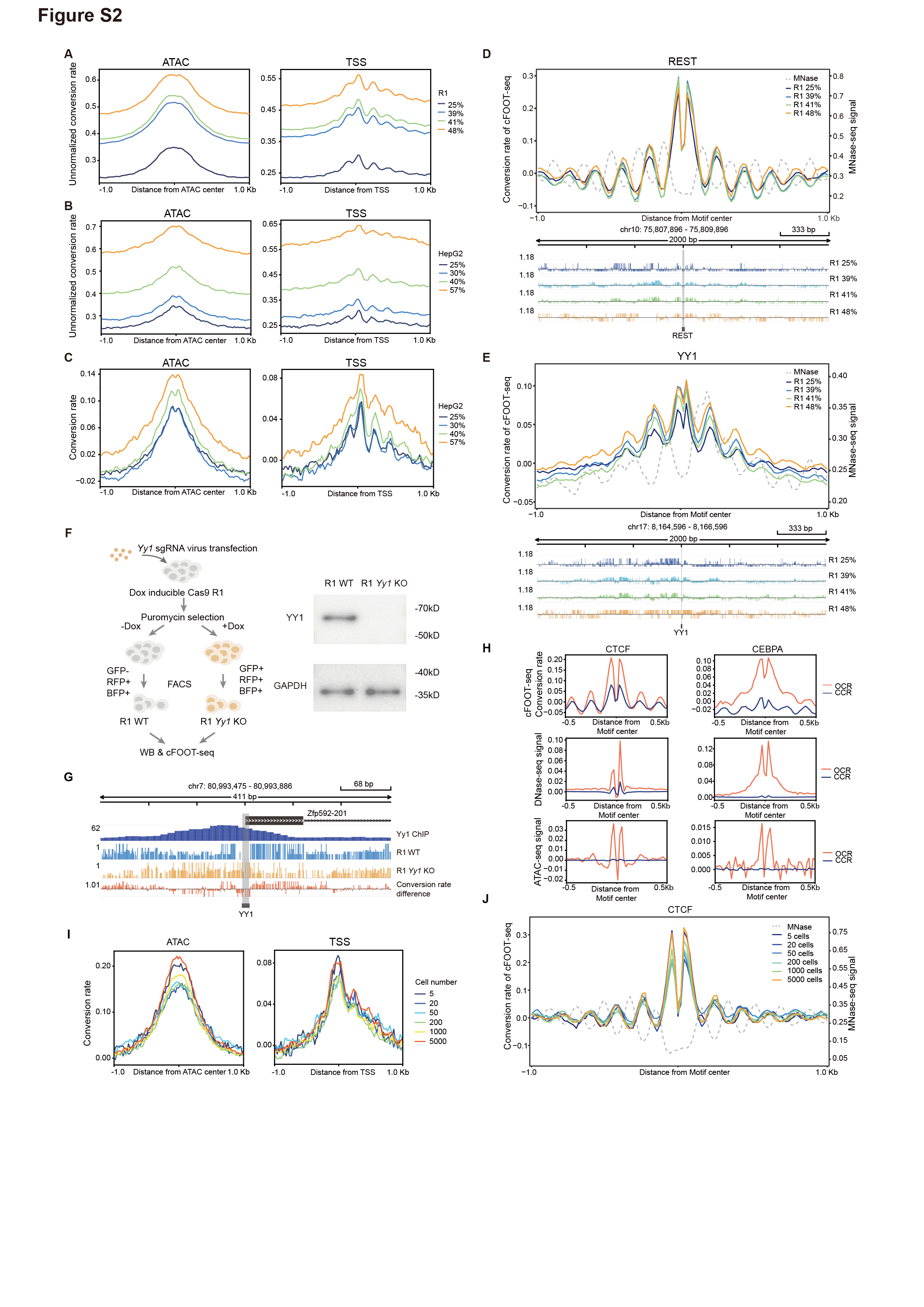
**

**Figure S2: cFOOT-seq maps chromatin accessibility, nucleosome occupancy, and TF footprint, related to Figure 1.**

**A.** Unnormalized average conversion rates around ATAC-seq peak center (left) and transcription start sites (TSS) (right) in R1 cells with increasing average genomic conversion rates.

**B.** Unnormalized average conversion rates around ATAC-seq peak center (left) and transcription start sites (TSS) (right) in HepG2 cells with increasing average genomic conversion rates.

**C.** Normalized average conversion rates around ATAC-seq peak center (left) and transcription start sites (TSS) (right) in HepG2 cells with increasing average genomic conversion rates.

**D-E.** Average profiles of normalized DNA conversion rates of cFOOT-seq and MNase-seq signal around all REST (E) or YY1 (F) binding sites defined by ChIP-seq aligned to their respective motifs (top), and distribution of DNA conversion rates at a representative genomic region flanking the CTCF or YY1 motif (bottom) in R1 cells. The pattern of nucleosome positioning as indicated by MNase-seq is shown with a grey dashed line. The profiles indicate binding events at motif centers and nucleosome patterns around REST or YY1 binding sites, showing samples with increasing average genomic conversion rates.

**F.** Schematic of *Yy1* KO R1 cell collection through FACS after Dox-induced expression of Cas9 (left). Western blot analysis of YY1 protein levels in cells with or without Dox induction, using antibodies against YY1 and GAPDH (right).

**G.** Distribution of DNA conversion rates around a representative YY1 binding site in R1 wt (33%) and *Yy1* KO (32%) cells. The YY1 ChIP-seq track (purple) shows the YY1 binding sites, and the position of the YY1 motif is indicated. cFOOT-seq conversion rates for R1 wt (blue) and R1 *Yy1* KO (orange) cells are shown. The conversion rate difference track (red) represents the difference in conversion rates between R1 w t and R1 *Yy1* KO cell, reflecting changes in TF binding at the YY1 motif

**H.** Average signal profiles of cFOOT-seq (top), DNase-seq (middle), and ATAC-seq (bottom) in open chromatin regions (OCR) and closed chromatin regions (CCR) around the centers of all CTCF (left) or CEBPA (right) motif center(±0.5kb).

**I.** Normalized DNA conversion rates around ATAC-seq peak center, TSS (±0.1kb) in R1 samples with low input cell numbers (5-5000).

**J.** Average profiles of normalized DNA conversion rates of cFOOT-seq and MNase-seq signal around CTCF binding sites aligned to CTCF motifs in R1 samples with low input cell numbers (5-5000).


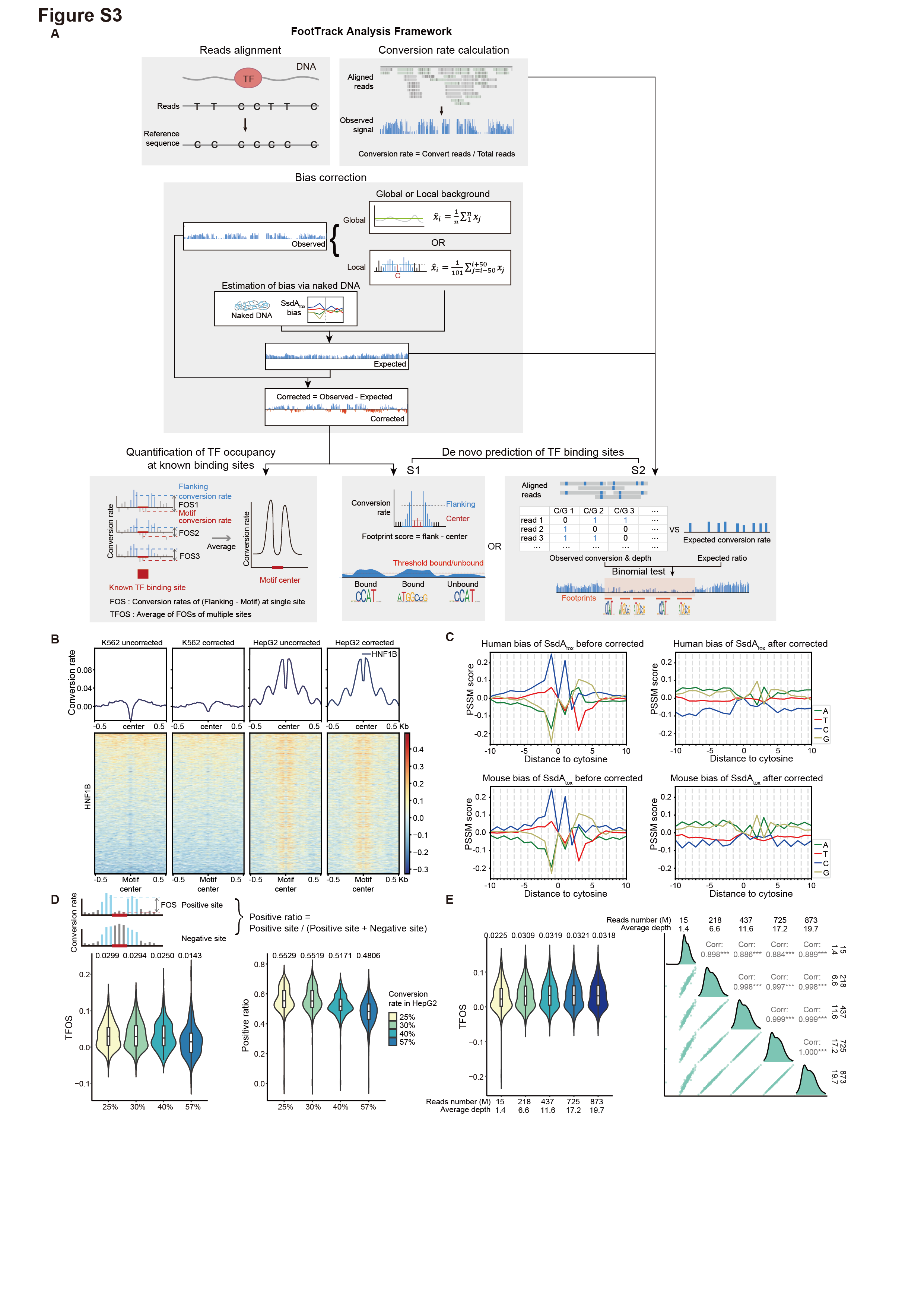


**Fig.S3: FootTrack analysis framework and performance of cFOOT-seq under different conditions, related to Figure 2.**

**A.** Analysis framework for cFOOT-seq processing, including read alignment, C/T conversion calling, bias correction, and downstream analysis. Bias correction is performed using two modes: global background (average conversion rate across the entire genome) and local background (average conversion rate within a ±50 bp window around each base). The corrected conversion rate is calculated by subtracting the expected from the observed conversion rate. The framework supports two primary analyses: (1) Quantification of TF occupancy at known binding sites, (2) De novo prediction of TF binding sites using strategy S1 (calculating footprint scores directly from corrected data) and S2 (predicting footprints using binomial statistical tests)

**B.** Average profiles and heatmaps of normalized DNA conversion rates around HNF1B binding sites in K562 and HepG2 cells, comparing the data before and after bias correction.

**C.** Sequence bias of SsdA_tox_ on naked DNA of human and mouse before and after bias correction. Each panel shows the position-specific scoring matrix (PSSM) scores for nucleotides A, T, C, and G across positions from -10 bp to +10 bp relative to cytosine.

**D.** Schematic illustration of the calculation of the positive ratio of FOS (top), along with violin plots showing TFOS scores (left) and the proportion of sites with positive FOS for each TF (right) under different genomic conversion rates in HepG2 cells using cFOOT-ATAC-seq. The conversion rates are color-coded and labeled at the bottom of each plot. The median values of TFOS and FOS positive ratio are shown above each violin plot.

**E.** Violin plots (left) and correlation plots (right) showing TFOS at various sequencing depths in HepG2 cells. The median TFOS values for all tested TFs in HepG2 is indicated above each violin plot. The total sequencing amount and average reads depth for each condition is listed for the corresponding plots.


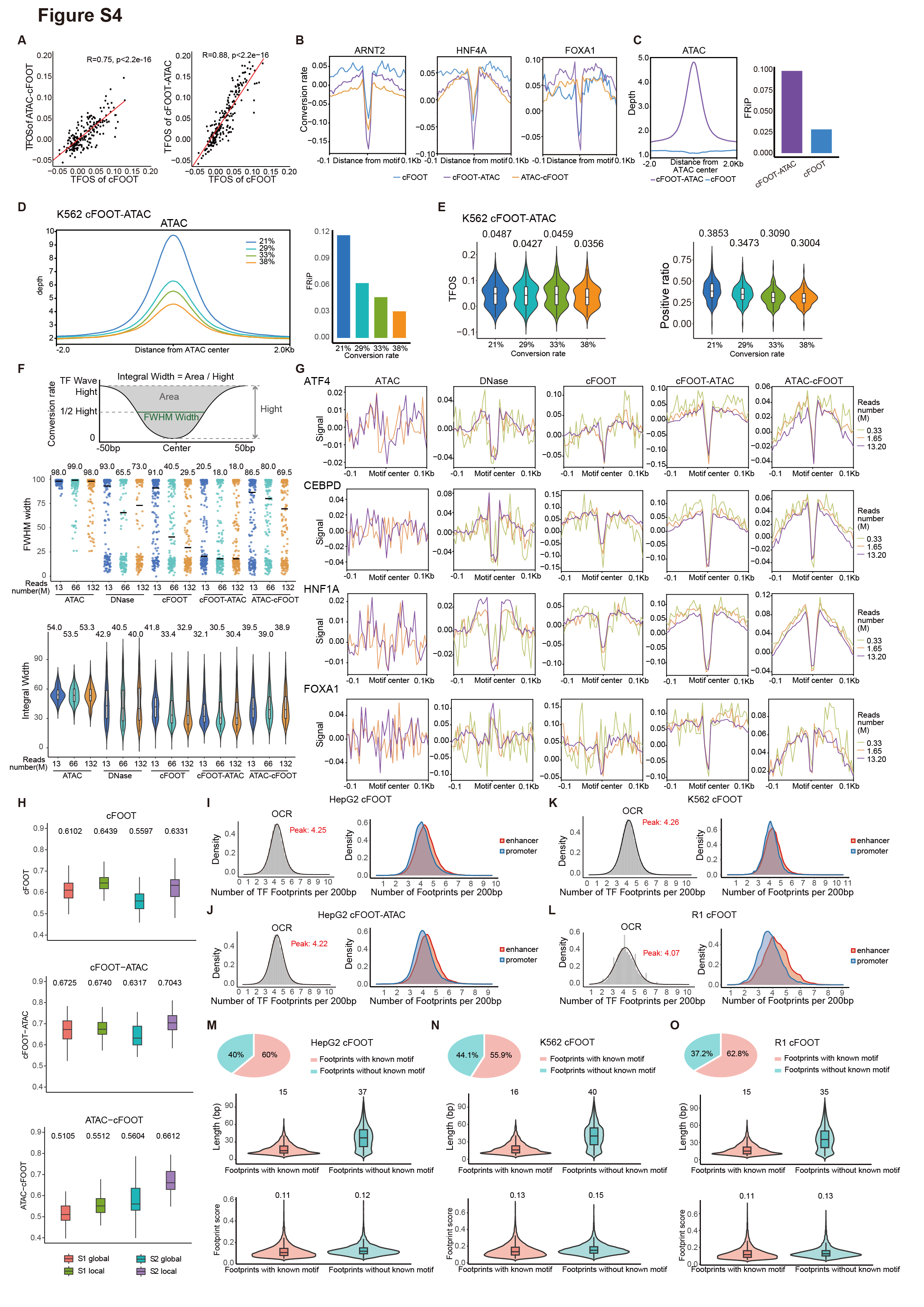


**Fig.S4 cFOOT-seq combined with ATAC-seq provide high-resolution and sensitive view of genomic TF occupancy, related to Figure 2.**

**A.** Scatter plot showing the correlation between TFOS differences of cFOOT-seq (30%) and ATAC-cFOOT-seq (33%) (R=0.75, p < 2.2e-16), cFOOT-seq (30%) and cFOOT-ATAC-seq (36%) (R=0.88, p < 2.2e-16) for each transcription factor (TFOS -0.05~0.20) in HepG2 cells.

**B.** Normalized average conversion rates around ±0.1 kb of ARNT2 (left), HNF4A (middle), and FOXA1 (right) motifs in HepG2 cells, comparing conversion rates from cFOOT-seq, cFOOT-ATAC-seq, and ATAC-cFOOT-seq methods.

**C.** Comparison of cFOOT-ATAC-seq and cFOOT-seq methods in HepG2 cells showing read depth around the ATAC-seq peak center (left) and FRiP values (right). Data for all conditions were obtained by extracting 10 million reads.

**D.** Comparison of cFOOT-ATAC-seq (29%) depth at ATAC-seq peak center with different conversion rates in K562 cells (left), and the corresponding FRiP values (right). Data for all conditions were obtained by extracting 10 million reads.

1. Violin plots showing TFOS scores (left) and the proportion of sites with positive FOS for each TF (right) under different genomic conversion rates in K562 cells using cFOOT-ATAC-seq. The conversion rates are color-coded and labeled at the bottom of each plot. The median values of TFOS and positive FOS ratio for all tested TFs are indicated above each violin plot.

**F.** Schematic representation (top) of the calculation for integral width and full-width half-maximum (FWHM) width of TF footprints. The scatter plot (middle) and violin plot (bottom) show the distribution of FWHM width and integral width, respectively, illustrating the resolution of ATAC-seq, DNase-seq, cFOOT-seq, cFOOT-ATAC-seq, and ATAC-cFOOT-seq methods in detecting 204 HepG2 TF footprints across different sequencing depths. The median values of FWHM width and integral width of each tested method are shown above the graphs.

**G.** Normalized average conversion rates around ±0.1 kb of ATF4, CEBPD, HNF1A, FOXA1 motif center in HepG2 cells. The plot compares the ability of ATAC-seq, DNase-seq, cFOOT-seq, cFOOT-ATAC-seq, and ATAC-cFOOT-seq methods to detect these four TF footprints under different reads number (0.33 M, 1.65 M, 13.20 M). The signal of ATAC-seq with 0.33 M is not available due to low reads number

**H.** Area under the ROC curve (AUC) for both strategies (S1 and S2) using local and global background modes. Results are shown for cFOOT-seq (left), cFOOT-ATAC-seq (middle), and ATAC-cFOOT-seq (right) of HepG2.

**I-L.** Density distributions of de novo predicted footprints (per 200 bp) by FootTrack in open chromatin regions (OCRs, defined by ATAC-seq), with comparisons between promoter-associated and enhancer-associated OCRs in HepG2 cFOOT-seq (I), HepG2 cFOOT-ATAC-seq (J), K562 cFOOT-seq (K), and R1 cFOOT-seq (L).

**M-O.** Pie charts show the proportions of footprints in OCRs with or without known motifs. Violin plots display the distributions of footprint lengths (middle) and footprint scores (bottom) for each category in HepG2 cFOOT-seq (M), K562 cFOOT-seq (N), and R1 cFOOT-seq (O).


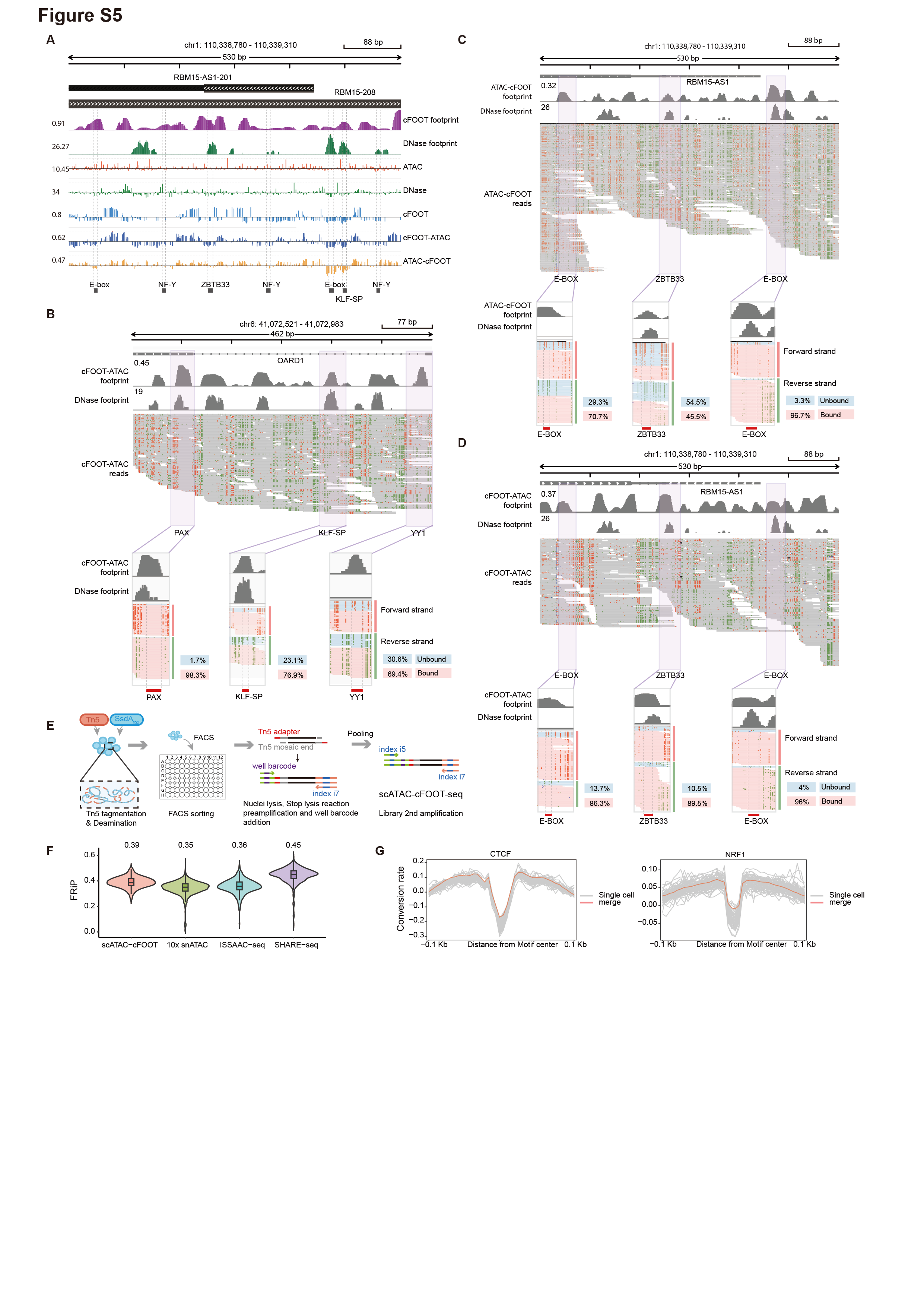


**Figure S5: Single-molecule and single-cell analysis of cFOOT-seq combinational methods, related to Figure 2.
A.** IGV browser graphics showing profiles of cFOOT-seq, cFOOT-ATAC-seq, ATAC-cFOOT-seq, DNase-seq, and ATAC-seq at a representative site (chr1: 110,338,780–110,339,310) in HepG2 cells. The "cFOOT footprint" track shows footprint scores derived from cFOOT-seq data, while the "DNase footprint" track shows footprint probabilities from DNase-seq data. The five tracks below display the distribution of corrected cutting events for ATAC-seq and DNase-seq, along with the corrected conversion rates for cFOOT-seq, cFOOT-ATAC-seq, and ATAC-cFOOT-seq. The black labels at the bottom highlight motifs identified in ChIP-seq peaks, corresponding to TF binding sites
**B.** IGV browser visualization showing the single-molecule profile of cFOOT-ATAC-seq at a representative site (chr6: 41,072,521–41,072,983) in HepG2 cells. The "cFOOT-ATAC footprint" track represents footprint scores calculated from cFOOT-ATAC-seq data, while the "DNase-seq footprint" track shows footprint probabilities derived from DNase-seq data. The " cFOOT-ATAC reads" track displays individual mapped reads in this region. Both forward and reverse strands are shown for the three TF motifs (PAX, KLF-SP, and YY1), and categorized into bound and unbound states based on conversion rates at each motif
**C-D.** IGV browser visualization showing the single-molecule profile of of ATAC-cFOOT-seq data (C) and cFOOT-ATAC data (D) at a representative site (chr1: 110,338,780–110,339,310) in HepG2 cells. The " cFOOT-ATAC footprint" track represents footprint scores calculated from cFOOT-ATAC-seq data, while the "DNase footprint" track shows footprint probabilities derived from DNase-seq data. The " cFOOT-ATAC reads" track displays individual mapped reads in this region. Both forward and reverse strands are shown for the three TF motifs (two E-boxes, and ZBTB33), and categorized into bound and unbound states based on conversion rates at each motif

**E.** Schematic of the experimental workflow for scATAC-cFOOT-seq, including Tn5 fragmentation, deamination, FAC sorting, barcoding, and pooling for library construction.
**F.** Violin plots showing the distribution of FRiP (fraction of reads in peaks) for single cells in scATAC-cFOOT-seq, 10x snATAC-seq, ISSAAC-seq and SHARE-seq of K562.
**G.** Plot showing the conversion rate at aggregated CTCF (left) and NRF1 (right) binding motifs in individual single cells (gray lines) and the merged data (red line) of K562.


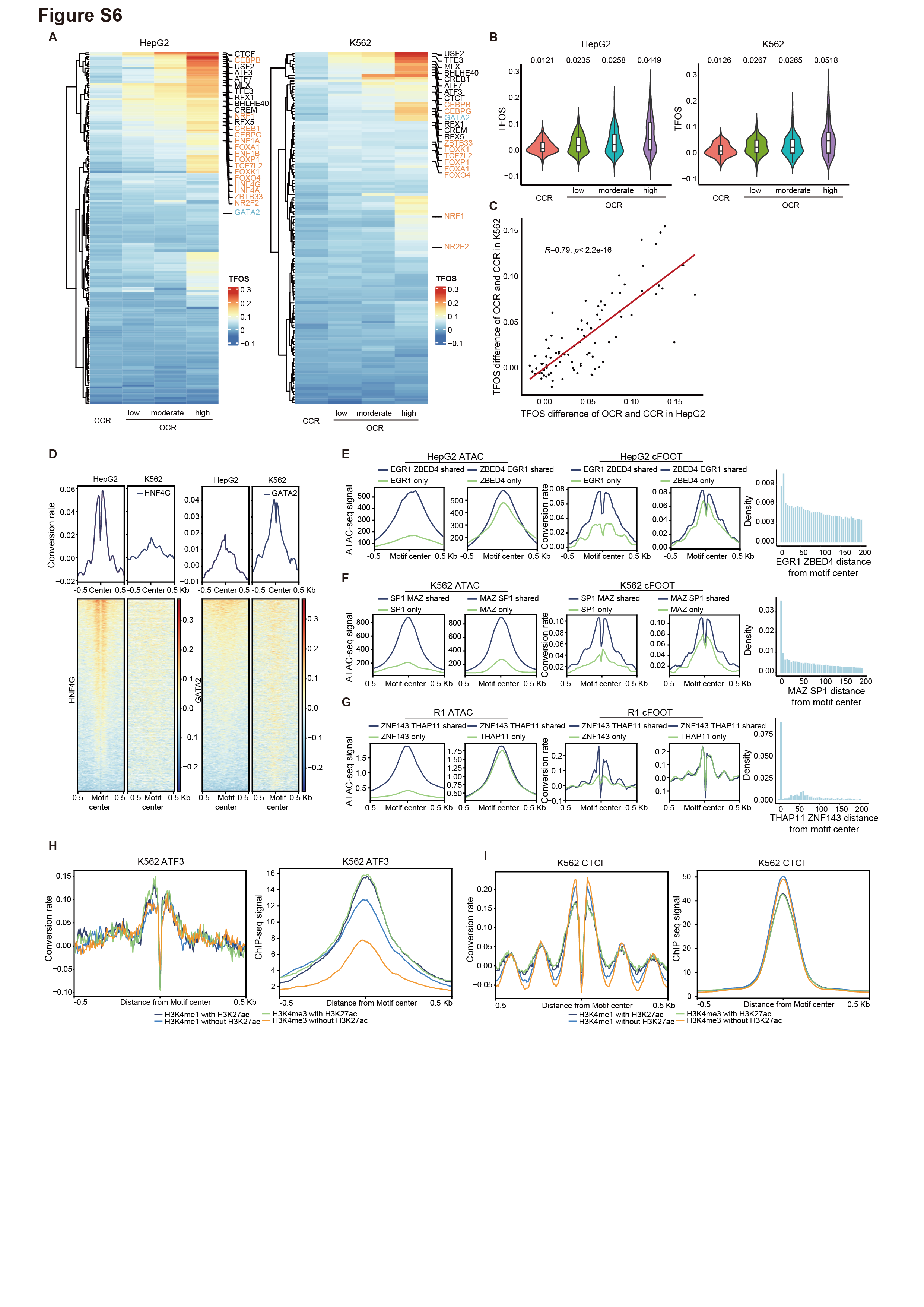


**Figure S6: cFOOT-seq quantitatively assess the impact of chromatin accessibility, histone modifications, and cofactors on TF occupancy, related to Figure 3.**

**A.** Heatmaps showing TFOS across different chromatin accessibility regions in HepG2 (left, 30%) and K562 (right, 30%) cells. Regions are categorized into closed chromatin regions (CCR) and low, moderate, and high open chromatin regions (OCR). TFs highlighted in orange indicate those with higher TFOS in OCR of HepG2, while TFs highlighted in blue indicate those with higher TFOS in OCR of K562. The color scale represents TFOS depth, with higher values in red and lower values in blue.

**B.** Violin plots illustrating the distribution of TFOS scores in different chromatin accessibility regions (CCR, low, moderate, high OCR) for HepG2 (left) and K562 (right) cells. The median TFOS values for each regioon are indicated above each violin plot.

**C.** Scatter plot showing the correlation between TFOS differences of OCR and CCR for each transcription factor in HepG2 and K562 cells. (n=89, R=0.79, p < 2.2e-16).

**D.** Average profiles and heatmaps displaying corrected DNA conversion rates around the motif centers for HNF4G and GATA2 binding sites defined by ChIP-seq in HepG2 and K562 cells. The differences in conversion patterns between HepG2 and K562 cells indicate cell type-specific flanking chromatin accessibility and TF occupancy around the motif centers for HNF4G and GATA2

**E.** Average profiles showing ATAC-seq reads depth (left) or corrected DNA conversion rates (middle) around ZBED4 and EGR1 binding sites in HepG2 cells, and the density distribution of distances between EGR1 and ZBED4 motif centers (right).

**F**. Average profiles showing ATAC-seq reads depth (left) or corrected DNA conversion rates (middle) around MAZ and SP1 binding sites in K562 cells, and the density distribution of distances between MAZ and SP1 motif centers (right).

**G**. Average profiles showing ATAC-seq reads depth (left) or corrected DNA conversion rates (middle) around ZNF143 and THAP11 binding sites in R1 cells, and the density distribution of distances between ZNF143 and THAP11 motif centers (right).

**H-I.** Average profiles showing corrected DNA conversion rate (left) and ChIP-seq signal (right) around ATF3 (**H**) and CTCF (**I**) binding sites in K562 cells. Binding sites were defined by ChIP-seq of each TF. Both cFOOT-seq and ChIP-seq signals are shown separately for regions marked by different combinations of histone modifications (H3K4me1^+^ H3K27ac^-^, H3K4me1^+^ H3K27ac^+^, H3K4me3^+^ H3K27ac^-^, and H3K4me3^+^ H3K27ac^+^) of K562 cells.

**
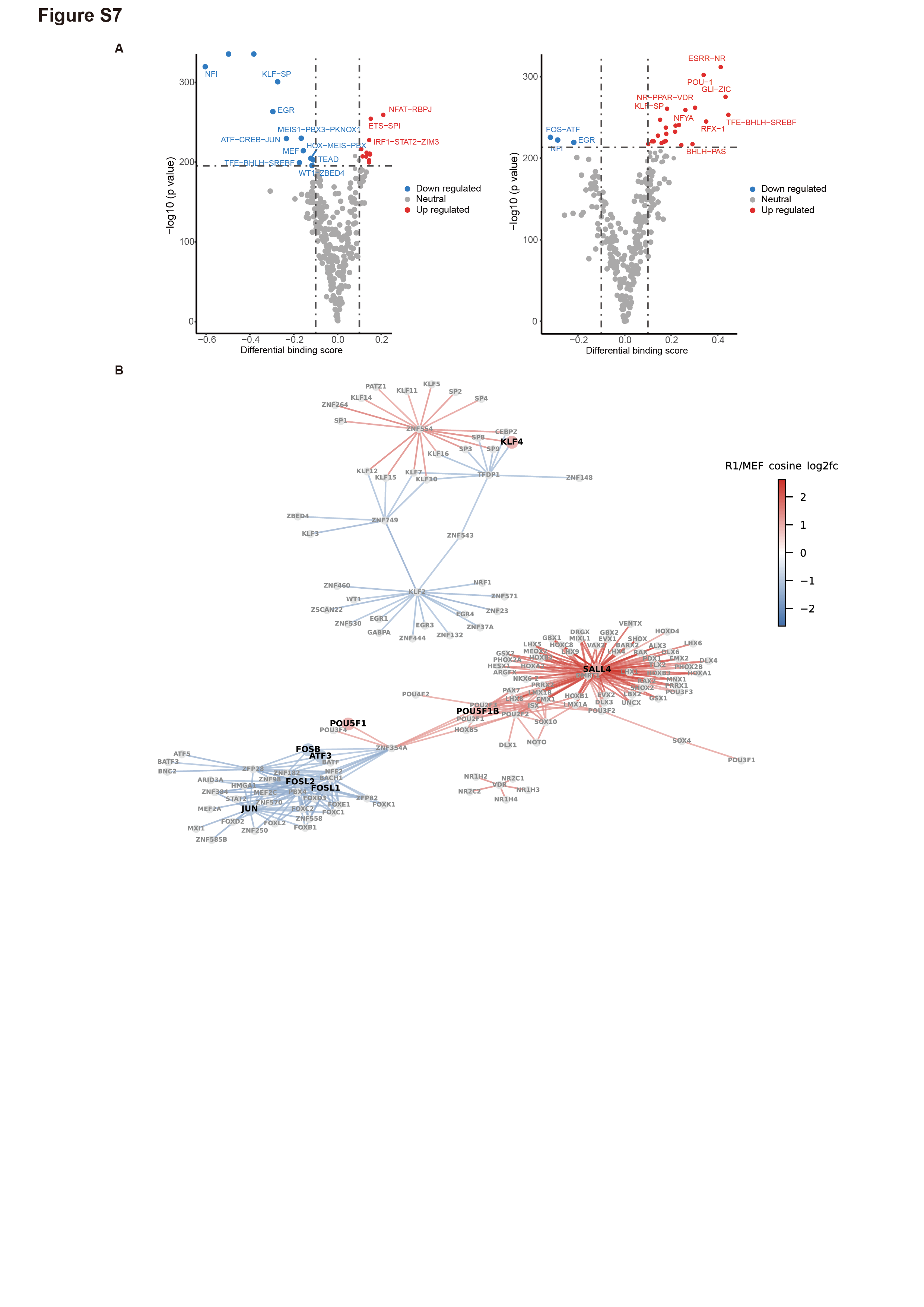
**

**Figure S7: Detailed analysis of TF binding differences in MEF and R1, related to Figure 4.**

**A**. Volcano plots showing FootTrack-predicted TF clusters with differential TF binding scores between MEF and R1 cells in MEF open regions (left) and R1 open regions (right). TFs with significantly upregulated footprint scores in R1 cells are highlighted in red, while those significantly downregulated in R1 are highlighted in blue.

**B**. Transcription factor co-occurrence network changes Between MEF and R1 Cells. This network diagram illustrates the co-occurring transcription factor (TF) pairs with edges colored based on the cosine log2 fold change (log2fc) between R1 and MEF cells. Nodes representing TFs specific to R1 are highlighted in red, while those specific to MEF are highlighted in blue, indicating differential TF associations unique to each cell type.


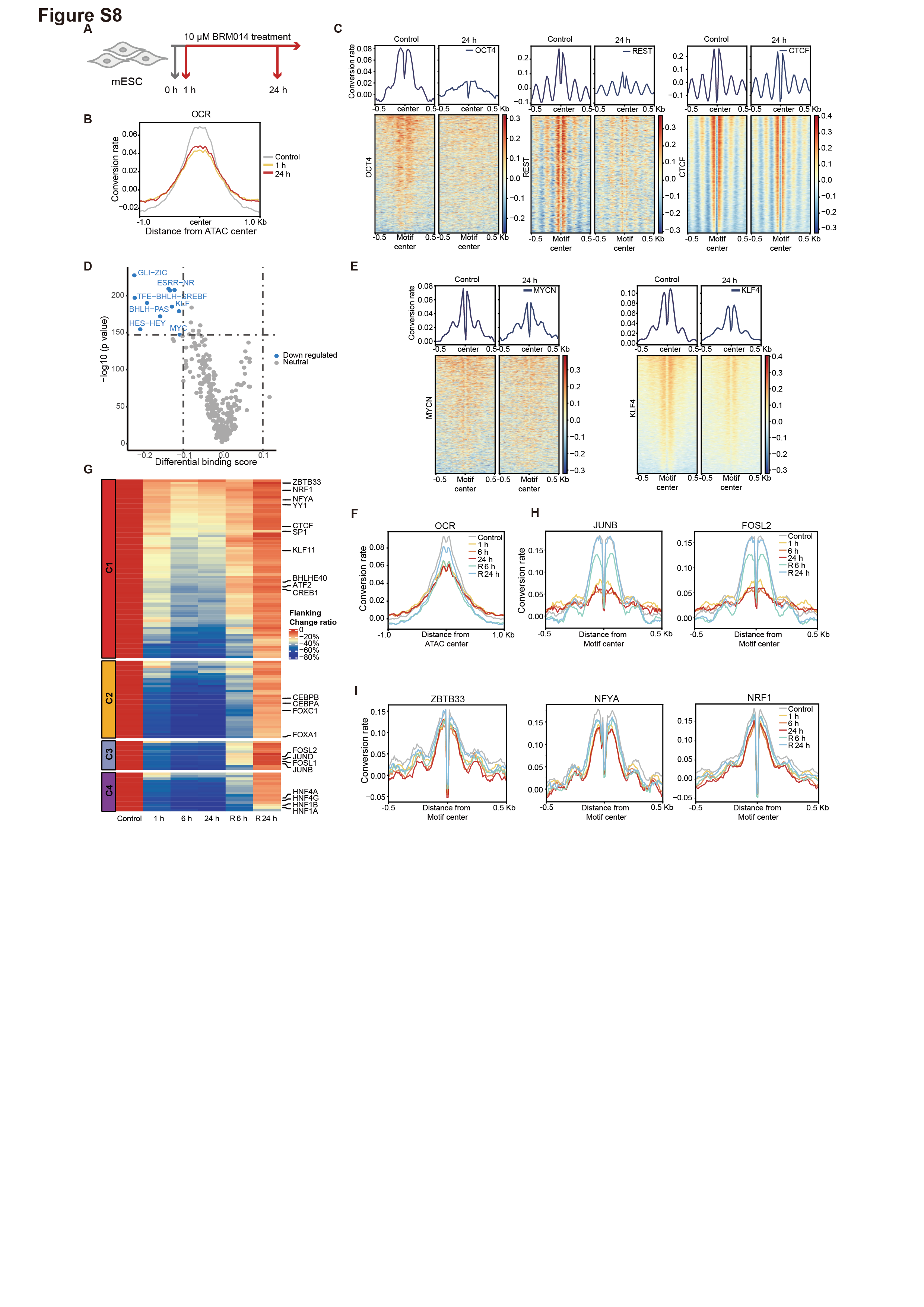


**Figure S8: cFOOT-seq depicts dynamics of nucleosome organization and TF occupancy in response to inhibition of SWI/SNF, related to Figure 5.**

**A.** Schematic representation of BRM014 treatments on mESCs (OG2 cells).

**B.** Average profile showing normalized DNA conversion rates around OCR defined by ATAC-seq in mESCs (OG2 cells) treated with BRM014 at 0 h (control, 39%), 1 h (40%), and 24 h (38%).

**C.** Average profiles and heatmaps showing DNA conversion rates around OCT4, REST, and CTCF binding sites under control conditions and after 24 h BRM014 treatment. OCT4 and REST sites show decreased chromatin accessibility and altered TF footprints, while CTCF sites exhibit minimal change.

**D.** Volcano plots showing FootTrack-predicted TF clusters with differential TF binding scores between control and 24 h BRM014-treated OG2 cells at open regions. TFs with downregulated scores are shown in blue, indicating decreased binding activity.

**E.** Average profiles and heatmaps of conversion rates around MYCN and KLF4 binding sites defined by ChIP-seq in OG2 cells treated with BRM014 for 0 h (control) and 24 h, aligned to motif centers and ranked by average conversion rates.

**F.** Average profiles of DNA conversion rates around OCR illustrating changes in chromatin accessibility following different durations of BRM014 treatment and recovery in HepG2 cells.

**G.** Heatmap showing the dynamic changes in the flank accessibility of 4 categories of TFs responding to BRM014 as defined by TFOS change ratio, with the left label marked the consensus clusters derived from TFOS (as shown in 5C), and the colors represent the proportion of flank accessibility change relative to the 0 h untreated baseline.

**H-I.** Average profiles of DNA conversion rates around TF binding sites of JUNB and FOSL2 (H), ZBTB33, NFYA and NRF1 (I) under control, 1 h, 6 h, and 24 h BRM014 treatments, as well as 6 h and 24 h recovery after 24 h BRM014 treatment. The profiles show changes in chromatin accessibility and TF footprints across different treatment conditions.


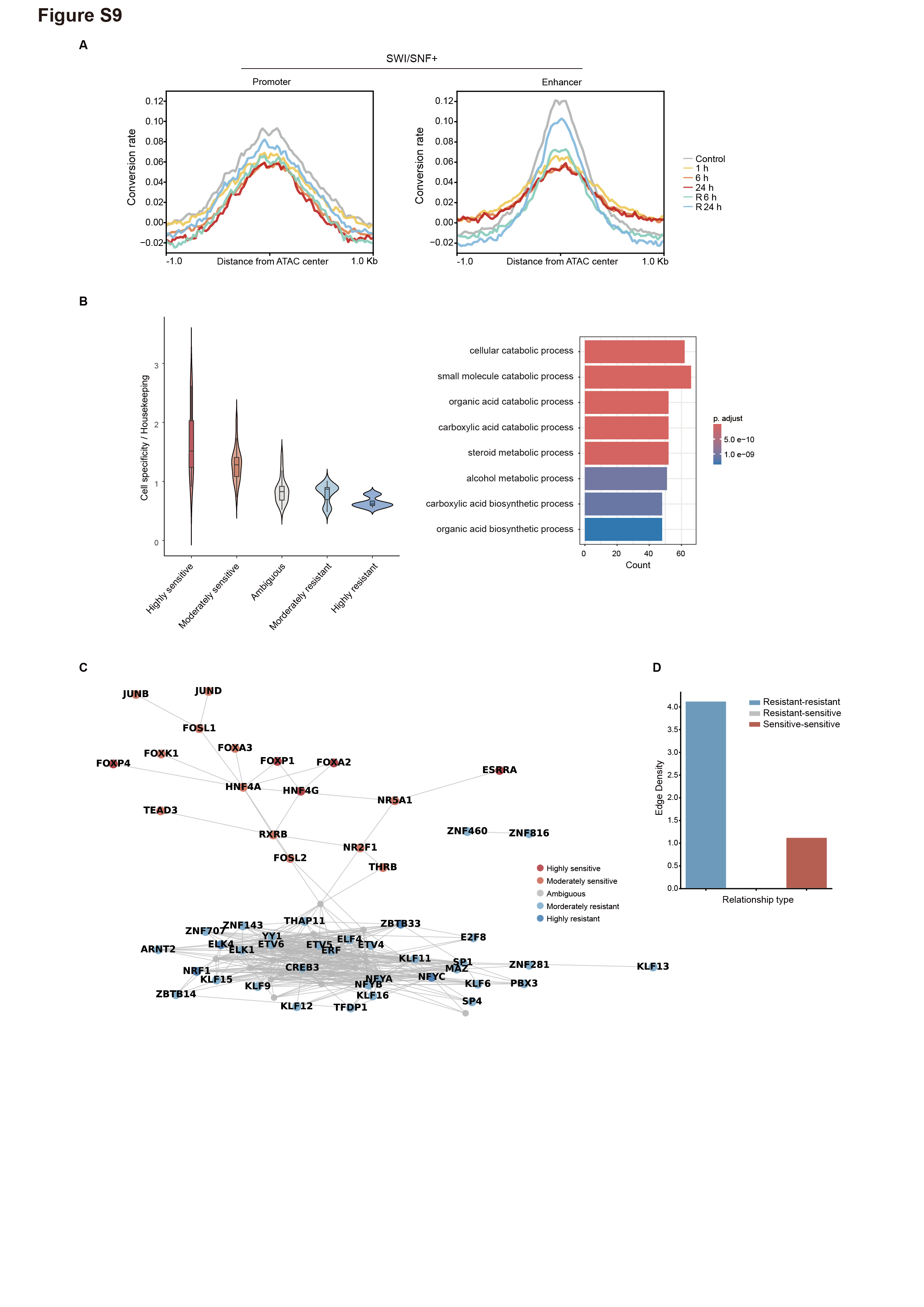


**Figure S9: Definition of TF dependency on SWI/SNF reveals spatial organization rule of TFs, related to Figure 6.**

**A.** Average profiles of DNA conversion rates in promoter (left) and enhancer (right) around SWI/SNF^+^ OCR regions, illustrating changes in chromatin accessibility following different durations of BRM014 treatment and recovery.

**B.** Violin plot showing, for each TF class, the ratio between the proportion of TF binding at cell-specific gene promoters and that at housekeeping gene promoters (left). GO enrichment of HepG2-specific genes (right).

**C.** This network diagram displays the co-occurrence of transcription factor (TF) pairs within the promoter regions of HepG2 cells. Each node represents a different TF, with the color coding indicating varying levels of sensitivity or resistance. Edges between nodes indicate potential co-occurrence relationships among the TFs

**D.** The bar graph displays the edge density in transcription factor co-occurrence network within the promoter regions of HepG2 cells among transcription factor pairs, categorized by their sensitivity to BRM014. The categories include interactions between TF pairs that are both resistant (blue bar), one resistant and one sensitive (grey bar), and both sensitive (red bar).
