# Supplementary material for: Genome-wide investigation of transcription factor footprints and dynamics using cFOOT-seq
Source: Protein Cell. 2025 Aug 4;16(11):932–52. doi: 10.1093/procel/pwaf071 (PMC12698189; doi:10.1093/procel/pwaf071)
Supplement: pwaf071_Supplementary_Materials [file pwaf071_supplementary_materials.zip › pwaf071_suppl_Supplementary_Table_S6.docx]

**Table S6. Oligo sequence, related to Methods**

| **Name** | **Sequence** | **Description** |
| --- | --- | --- |
| sgYy1_01 | TATGTAGTCGTCGTCGCCGC | sgRNA used to generate YY1 KO R1 cell |
| sgYy1_02 | CTACATCGCCACGGACGGCT | sgRNA used to generate YY1 KO R1 cell |
| sgYy1_03 | AGCGGCTGCAGCGCGATCAT | sgRNA used to generate YY1 KO R1 cell |
| Substrate_ACTCGCC_F | 5'6-FAM-AATATAATATAATAACTCGCCATAATTTTAATTAAT | Substrate_ACTCGCC_F and Substrate_ACTCGCC_R are used to anneal deaminase substrate with ACTCGCC |
| Substrate_ACTCGCC_R | ATTAATTAAAATTATGGCGAGTTATTATATTATATT |  |
| Substrate_AC_F | 5'6-FAM-AATATAATATAATAACATAATTTTAATTAAT | Substrate_AC_F and Substrate_AC_R are used to anneal deaminase substrate with AC |
| Substrate_AC_R | ATTAATTAAAATTATGTTATTATATTATATT |  |
| Substrate_TC_F | 5'6-FAM-AATATAATATAATATCATAATTTTAATTAAT | Substrate_TC_F and Substrate_TC_R are used to anneal deaminase substrate with TC |
| Substrate_TC_R | ATTAATTAAAATTATGATATTATATTATATT |  |
| Substrate_GC_F | 5'6-FAM-AATATAATATAATAGCATAATTTTAATTAAT | Substrate_GC_F and Substrate_GC_R are used to anneal deaminase substrate with GC |
| Substrate_GC_R | ATTAATTAAAATTATGCTATTATATTATATT |  |
| Substrate_CC_F | 5'6-FAM-AATATAATATAATACCATAATTTTAATTAAT | Substrate_CC_F and Substrate_CC_R are used to anneal deaminase substrate with CC |
| Substrate_CC_R | ATTAATTAAAATTATGGTATTATATTATATT |  |
| Tn5 Primer D | TGGTAGAGAGGGTGAGATGTGTATAAGAGACAG | Tn5 Primer D annealed with Tn5 ME to prepare Tn5 adapter with  cytosine-free 5’ overhang |
| Tn5 ME | 5‘-phos-CTGTCTCTTATACACATCT-NH2-3’ |  |
| i5_bridge primer | ACACTCTTTCCCTACACGACGCTCTTCCGATCTTGGTAGAGAGGGTGAGATGTGTATAAGAGACAG | together with universal i7 primers to do the first amplification of DNA from cFOOT-ATAC or ATAC-cFOOT, to add the sequence complementary with i5 |
| C-DNA / 5mC-DNA | GAATTCTTGCAGCACTAGTGCATCTATAAGTTATCTCAAATCAAGAAATCAGTCTAATGAGAATTTCAATAACTTCAGCAATTTAAGCTGCATGCATCAGTGTCATCGTTATTTTTTTTTTGAGACGTAGTCATGCTCTGTTGCTGAGTCTGCAGTACAGTGACGAGATATCGACTCAGCACAACATCTGCATCACATGTTCAAGCGATTCTCATGCTTCAGCTTGCAGAGTAGCTGTCACTACAGACACTGAGCAGCATGCGTGACTAATTTTTGTATTTTTAGTAGAGAGTGCATTTCGTCATGTTGTACAGTCTAGTTTCAAACTCATGACTTCAGTTGATCTAACTGACACGATCTCAGAATTTACTGTCATTACAGTACTGTCACACAGTGACAGTCATTTTTCTTAATTTTTAAAAATATTAAAGTTTTATCTCATTCGTGTTGAAGCATATTCGTGATTTAAAAGTTGCAAAG | DNA template for prepairing substrate with 5mC or C by PCR |
